# Supplementary material for: Strengthening the Reporting of Genetic Risk Prediction Studies: The GRIPS Statement
Source: PLoS Med. 2011 Mar 15;8(3):e1000420. doi: 10.1371/journal.pmed.1000420 (PMC3058100; doi:10.1371/journal.pmed.1000420)
Supplement: Text S1 — Strengthening the Reporting of Genetic RIsk Prediction Studies (GRIPS): Explanation and Elaboration. (1.44 MB DOC) [file pmed.1000420.s001.doc]

**Strengthening the Reporting of Genetic Risk Prediction Studies (GRIPS): Explanation and Elaboration**

A. Cecile J.W. Janssens^1,*^, John P.A. Ioannidis^2,3,4,5,6^, Sara Bedrosian^7^, Paolo Boffetta^8,9^, Siobhan M. Dolan^10^, Nicole Dowling^7^, Isabel Fortier^11^, Andrew N. Freedman^12^, Jeremy M. Grimshaw^13,14^, Jeffrey Gulcher^15^, Marta Gwinn^7^, Mark A. Hlatky^16^, Holly Janes^17^, Peter Kraft^12^, Stephanie Melillo^7^, Christopher J. O’Donnell^19,20^, Michael J. Pencina^21^, David Ransohoff^22^, Sheri D. Schully^12^, Daniela Seminara^12^, Deborah M. Winn^12^, Caroline F. Wright^23^, Cornelia M. van Duijn^1^, Julian Little^24^, Muin J. Khoury^7^

^1^ Department of Epidemiology, Erasmus University Medical Center, Rotterdam, The Netherlands.

^2^ Department of Hygiene and Epidemiology, University of Ioannina School of Medicine, Ioannina, Greece.

^3^ Biomedical Research Institute, Foundation for Research and Technology, Ioannina, Greece.

^4^ Department of Medicine, Tufts University School of Medicine, Boston MA, USA.

^5^ Center for Genetic Epidemiology and Modeling and Tufts CTSI, Institute for Clinical Research and Health Policy Studies, Tufts Medical Center, Boston MA, USA.

^6^ Stanford Prevention Research Center, Stanford University School of Medicine, Stanford CA, USA.

^7^ Office of Public Health Genomics, Centers for Disease Control and Prevention, Atlanta GA, USA.

^8^ The Tisch Cancer Institute, Mount Sinai School of Medicine, New York NY, USA.

^9^ International Prevention Research Institute, Lyon, France.

^10^ Department of Obstetrics & Gynecology and Women’s Health, Albert Einstein College of Medicine / Montefiore Medical Center, Bronx NY, USA.

^11^ Public Population Project in Genomics (P3G), Montreal, Quebec, Canada.

^12^ Division of Cancer Control and Population Sciences, National Cancer Institute, National Institutes of Health, Bethesda MD, USA.

^13^ Clinical Epidemiology Program, Ottawa Hospital Research Institute, Ottawa ON, Canada;

^14^ Department of Medicine, University of Ottawa, Ottawa ON, Canada.

^15^ deCODE Genetics, Reykjavik, Iceland.

^16^ Department of Health Research and Policy, Stanford University, Palo Alto CA, USA.

^17^ Fred Hutchinson Cancer Research Center, Vaccine and Infectious Disease Institute and Division of Public Health Sciences, Seattle WA, USA.

^18^ Department of Epidemiology, Harvard School of Public Health, Boston MA, USA.

^19^ National Heart, Lung and Blood Institute (NHLBI) and the NHLBI's Framingham Heart Study, Framingham MA, USA;

^20^ Cardiology Division, Massachusetts General Hospital, Harvard Medical School, Boston MA, USA.

^21^ Department of Biostatistics, Boston University, Boston MA, USA; Harvard Clinical Research Institute, Boston MA, USA.

^22^ University of North Carolina at Chapel Hill School of Medicine, Chapel Hill NC, USA.

^23^ PHG Foundation, Cambridge, United Kingdom.

^24^ Department of Epidemiology and Community Medicine, University of Ottawa, Ottawa ON, Canada.

*** Corresponding author**

A. Cecile J.W. Janssens, Erasmus University Medical Center, Department of Epidemiology, PO Box 2040, 3000 CA Rotterdam, the Netherlands. Email: a.janssens@erasmusmc.nl; Telephone: +31-10-7044214; Fax: +31-10-7044657.

Running head: GRIPS statement: Explanation & Elaboration

**Summary Points**

- The rapid and continuing progress in gene discovery for complex diseases is fuelling interest in the potential application of genetic risk models for clinical and public health practice.
- The number of studies assessing the predictive ability is steadily increasing, but they vary widely in completeness of reporting and apparent quality.
- Transparent reporting of the strengths and weaknesses of these studies is important to facilitate the accumulation of evidence on genetic risk prediction.
- A multidisciplinary workshop sponsored by the Human Genome Epidemiology Network developed a checklist of 25 items recommended for strengthening the reporting of Genetic RIsk Prediction Studies (GRIPS), building on the principles established by prior reporting guidelines.
- These recommendations aim to enhance the transparency, quality and completeness of study reporting, and thereby to improve the synthesis and application of information from multiple studies that might differ in design, conduct or analysis.

**Introduction**

The advent of genome-wide association studies has accelerated the discovery of novel genetic markers, in particular single nucleotide polymorphisms (SNPs) that are associated with risk for common complex diseases. Technological developments in large-scale genomic studies, such as whole genome sequencing, will facilitate the discovery of novel of common SNPs, as well as of rare variants, copy number variations, deletions/insertions, structural variations (e.g., inversions), and epigenetic effects that influence the regulation of gene expression. These developments are fuelling interest in the translation of this basic knowledge to health care practice. Knowledge about genetic risk factors may be used to target diagnostic, preventive and therapeutic interventions for complex disorders based on a person’s genetic risk, or to complement existing risk models based on classical non-genetic factors such as the Framingham risk score for cardiovascular disease. Implementation of genetic risk prediction in health care requires a series of studies that encompass all phases of translational research [1,2], starting with a comprehensive evaluation of genetic risk prediction.

Genetic risk prediction studies typically concern the development and/or evaluation of models for the prediction of a particular health outcome, but there is considerable variation in their design, conduct and analysis. Genetic risk models most frequently predict risk of disease, but they are also being investigated for the prediction of prognostic outcome, treatment response or treatment side effects. Risk prediction models are used in research and clinical settings to classify individuals into homogeneous groups e.g., for randomization in clinical trials and for targeting preventive or therapeutic interventions. The main study designs are cohort, cross-sectional or case-control. The genetic risk factors often are SNPs, but other variants such as insertions/deletions, haplotypes and copy number variations can be included as well. The risk models are based on genetic variants only, or include both genetic and non-genetic risk factors. Risk prediction models are statistical algorithms, which can be simple genetic risk scores (e.g., risk allele counts), or be based on regression analyses (e.g., weighted risk scores or predicted risks) or on more complex analytic approaches such as support vector machine learning or classification trees. Papers on genetic risk prediction vary as to whether they present the development of a risk model only, the validation of one or more risk models only, or both development and validation of a risk model [3]. Lastly, studies vary in the measures used to assess model performance. So far, assessments have nearly always included measures of discrimination, but hardly any considered calibration [3]. Recent studies have additionally assessed measures of reclassification, despite debate on the appropriate use and interpretation of these measures [4,5].

So far most genetic prediction studies have shown that the predictive performance of genetic risk models is poor, with some exceptions such as those for age-related macular degeneration, hypertriglyceridemia and Crohn’s disease [6–8]. While the poor performance is most likely due to the low number of variants that have been definitely linked to a phenotype to date, many publications lack sufficient details to judge methodological or analytic aspects. Information that is often missing includes details in the description of how the study was designed and conducted (e.g., how genetic variants were selected, how risk models or genetic risk scores were constructed and how risk categories were chosen), or how the results should be interpreted. An appropriate assessment of the study’s strengths and weaknesses is not possible without this information. With increasing numbers of discovered genetic markers that can be used in future genetic risk prediction studies, it is crucial to enhance the quality of the reporting of these studies, since valid interpretation could be compromised by the lack of reporting of key information. There is ample evidence that prediction research often suffers from poor design and biases, and these might have an impact also on the results of the studies and on models of disease outcomes based on these studies [9–11]. Although most prognostic studies published to date claim significant results [12,13], very few translate to clinically useful applications, in part because study findings resulted from chance, methodological biases or the inclusion of risk factors that had not been previously replicated. Just as for observational epidemiological studies [14], poor reporting complicates the use of the specific study for research, clinical, or public health purposes and the deficiencies also hamper the synthesis of evidence across studies.

Reporting guidelines have been published for various research designs [15] and these contain many items that are also relevant to genetic risk prediction studies. In particular, the guidelines for genetic association studies (STREGA) have relevant items on the assessment of genetic variants, and the guidelines for observational studies (STROBE) have relevant items about the reporting of study design. The guidelines for diagnostic studies (STARD) and those for tumor marker prognostic studies (REMARK) include relevant items about test evaluation, and the REMARK guidelines include relevant items about risk prediction [16–19]. However, none of these guidelines are fully suited to genetic risk prediction studies, an emerging field of investigations with specific methodological issues that need to be addressed, such as the handling of large numbers of genetic variants (from 10s to 10000s), which come with greater challenges and flexibility on how these can be dealt with in the analyses.

The main goal of this paper is to propose and justify a set of guiding principles for reporting results of Genetic RIsk Prediction Studies (GRIPS). To minimize confusion in the field, these recommendations build on prior reporting guidelines whenever possible. The intended audience for the reporting guideline is broad and includes epidemiologists, geneticists, statisticians, clinician scientists and laboratory-based investigators who undertake genetic risk prediction studies, as well as journal editors and reviewers who have to appraise the design, conduct and analysis of such studies. In addition, it includes 'users' of such studies who wish to understand the basic premise, design, and limitations of genetic prediction studies in order to interpret the results for their potential application in health care. These guidelines are also intended to ensure that essential data from genetic risk prediction studies are presented, which will facilitate information synthesis as part of systematic reviews and meta-analyses.

Finally, it is important to emphasize that these recommendations are guidelines only for how to report research; the recommendations do not prescribe how to perform genetic risk prediction studies. Nevertheless, we suggest that increased transparency of reporting might have a favorable effect on the quality of research, and thereby improve the translation into practice, as has been the case for the adoption of the CONSORT checklist in the reporting of randomized controlled trials [20].

**Development of the GRIPS Statement**

The GRIPS Statement was developed by a multidisciplinary panel of 25 risk prediction researchers, epidemiologists, geneticists, methodologists, statisticians and journal editors, seven of whom were also part of the STREGA initiative [17]. They attended a two-day meeting in Atlanta, GA, USA, in December 2009 sponsored by the Centers for Disease Control and Prevention on behalf of the Human Genome Epidemiology Network (HuGENet) [21]. Participants discussed a draft version of the checklist that was prepared and distributed prior to the meeting. This draft version was developed based on existing reporting guidelines, namely STREGA [17], REMARK [19], and STARD [18]. These were selected from all available guidelines (see www.equator-network.org) because of their focus on observational study designs and genetic factors (STREGA), prediction models (REMARK), and test evaluation (REMARK and STARD). Methodological issues pertinent to risk prediction studies were addressed in presentations during the meeting. Workshop participants revised the initial recommendations both during the meeting and in extensive electronic correspondence after the meeting. To harmonize our recommendations for genetic risk prediction studies with previous guidelines, we chose the same wording and explanations for the items wherever possible. Finally, we tried to maintain consistency with previous guidelines for the evaluation of risk prediction studies of cardiovascular diseases and cancer [2,22]. The final version of the checklist is presented in Table 1.

**Scope of the GRIPS Statement**

The GRIPS Statement is intended to maximize the transparency, quality and completeness of reporting on research methodology and findings in a particular study. Researchers can use the statement to inform their choice of study design and analyses, but the guidelines do not support or oppose the choice of any particular study design or method. For example, the guidelines recommend that the study population should be described, but do not specify which population is preferred in a particular study.

Items presented in the checklist are relevant for a wide array of observational risk prediction studies, because the checklist focuses on the main aspects in the design and analysis of risk prediction studies. GRIPS does not address randomized trials that may be performed to test risk models, nor does it specifically address decision analyses, cost-effectiveness analyses, assessment of health care needs or assessment of barriers to health care implementation [23]. Once the performance of a risk model has been established, these next steps towards implementation require further evaluation [24,25]. For the reporting of these studies, which go beyond the assessment of genetic risk models as such, additional requirements apply. However, proper documentation of genetic predictive research according to GRIPS might facilitate the translation of research findings into clinical and public health practice.

**How to use this paper**

This paper illustrates and elaborates on the items of the GRIPS Statement that are published in several journals. We modeled this Explanation and Elaboration document along the lines of those developed for other reporting guidelines [26–29]. The GRIPS Statement consist of 25 items grouped by article sections (title and abstract, introduction, methods, results and discussion). The discussion of each item in this paper follows a standardized format. First, we illustrate each item with one or more published examples of what we consider to be transparent reporting, drawn from the genetic risk prediction studies referenced in Table 2. Table or figure numbers in the examples refer to the tables and figures in the present manuscript, not the original article. Second, for each item, we explain in detail the rationale for its inclusion in the checklist. And third, we present details about each item that need to be addressed to ensure transparent reporting.

Frequently, papers about genetic risk prediction are conducted using data from multiple populations. Many studies have combined multiple datasets to develop the risk model, for example by obtaining controls and cases from different populations [7,30–32], or have derived risk models in multiple populations [33]. Studies may also use one or more populations to validate the model in independent samples. Readers need to be able to assess the similarities and differences among these populations in terms of the design of the study, selection of participants, data collection and analyses. Differences in the study designs and population characteristics that might impact the validity and generalizability of the findings should be reported. These may include ascertainment of participants, distributions of age, sex and ethnicity as well as the prevalence of risk factors, disease and co-morbidities [3]. Authors should describe any efforts made to harmonize the assessment methods, if these were different. The essential items that should be reported for each population are marked in Table 1.

Finally, genetic risk models may also be applied to predict other clinically relevant outcomes such as prognosis, treatment response and side effects of treatment. To improve the readability of the paper, the paper focuses on prediction of disease risk, but the items also apply to other health outcomes as well.

**The GRIPS Checklist**

For each checklist item shown in Table 1, this section provides examples of appropriate reporting from actual scientific articles of genetic risk models for diseases and health conditions, as well as an explanation of the importance and need for the item and helpful guidance about details that constitute transparent reporting.

TITLE and ABSTRACT

*Item 1: (a) Identify the article as a study of risk prediction using genetic factors. (b) Use recommended keywords in the abstract: genetic or genomic, risk, prediction.*

**Examples**. (Title) “Combining information from common type 2 diabetes risk polymorphisms improves disease prediction.” [34]

(Title) “Prediction model for prevalence and incidence of advanced age-related macular degeneration based on genetic, demographic, and environmental variables.” [6]

(Abstract) “Recent studies have evaluated whether incorporating nontraditional risk factors improves coronary heart disease (CHD) *prediction* models. This 1986–2001 US study aggregated the contribution of multiple single nucleotide polymorphisms into a *genetic* *risk* score (GRS) and assessed whether the GRS plus traditional risk factors predict CHD better than traditional risk factors alone.” [35]

(Abstract) “The degree to which currently known *genetic* variants can improve the *prediction* of CHD *risk* beyond conventional risk factors in this disorder was investigated.” [36]

**Explanation.** Public bibliographic databases have become an essential tool in knowledge synthesis and dissemination and a key source for identifying studies. To date, there is no single strategy that retrieves all or most papers on genetic risk prediction in these databases. Table 2 shows that the 24 studies of genetic risk prediction cited in this paper have used 17 different terms in their titles and one study made no reference to genetic factors at all [37]. PubMed Clinical Queries has implemented standardized search strategies for retrieving clinical prediction guides [38] and prognosis studies in general [39], but these are inefficient strategies to retrieve genetic risk prediction studies. The broad versions of both types of PubMed Clinical Queries were able to ascertain most of the listed papers, but at the same time many other studies not related to this topic (Table 2). To facilitate identification and indexing, authors are encouraged to exploit all three opportunities, namely title, abstract and Medical Subject Headings (MeSH terms), to help ensure the capture of the article in the clinical queries and routine PubMed searches.

In the abstract, authors should explicitly describe their work as a study of genetic risk prediction by using the three keywords: “genetic” (or “genomic”), “risk”, and “prediction”. These words do not need to be mentioned in a specific combination or order. If the report focuses on genetic risk prediction as a main objective, authors are advised to mention the keywords in the title. The use of the keyword “genetic” or “genomic” is particularly important because a variety of genetic variants exists, such as chromosomes, SNPs, haplotypes or copy number variations. It will be difficult to retrieve all relevant studies if authors only use the specific terminology and not a broad descriptor like “genetic variant”. Table 2 shows that the combination of the keywords was by far more specific in identifying the prediction studies that are cited in this paper as compared with the PubMed Clinical Queries. The use of these keywords is also essential when risk prediction is not the main objective of a study, for example when prediction analysis is part of genome-wide association studies [40]. To ensure that these articles are identifiable, authors should mention the prediction analysis in the abstract as well.

MeSH terms are another opportunity to identify an article as a study of genetic risk prediction, but this is often not under control of the author. The articles listed in Table 2 have been given a variety of MeSH terms and no single term or combination of terms would have retrieved all papers. To facilitate future synthesis of studies, we recommend that studies on this topic at least use the MeSH terms “genetic predisposition to disease”, “risk assessment” and “predictive value of tests”. These three terms are analogous to the keywords “genetic”, “risk” and “prediction”. Each MeSH term alone retrieved 18 of the articles listed in Table 2, and over 50,000 other articles (results not shown). The exact combination of the three MeSH terms did not retrieve any of these studies, but also only a little over 100 other papers in total. Consequently, assigning the three MeSH terms to genetic risk prediction studies potentially allows for a very specific search strategy to retrieve future articles.

INTRODUCTION

*Item 2: Explain the scientific background and rationale for the prediction study.*

**Example.** “Knowledge about genetic and epidemiologic associations with the leading cause of blindness among the elderly, age-related macular degeneration, has grown exponentially in recent years. Several genetic variants with strong and consistent

associations with AMD have recently been identified. We also know that in addition to age, ethnicity, and family history, there are modifiable factors: smoking, nutritional

antioxidants and omega-3 fatty acid intake, and overall and abdominal adiposity. However, it remains unknown whether all these genetic and environmental factors act independently or jointly and to what extent they as a group can predict the occurrence of age-related macular degeneration (AMD) or progression to advanced AMD from early and intermediate stages. Such information might be useful for screening those at high risk due to a positive family history or having signs of early or intermediate disease, among whom some progress to advanced stages of AMD with visual loss. Early detection could reduce the growing societal burden due to AMD by targeting and emphasizing modifiable habits earlier in life and recommending more frequent surveillance for those highly susceptible to the disease.” [6]

**Explanation.** The background should inform the reader what is already known on the topic, and what gaps in knowledge justify conducting the present study. Relevant background information should include, but is not limited to, the following two topics:

First, what is known about the role of genetic factors in the outcome of interest, and in particular about the genetic variants that are being considered for inclusion in the prediction model? Such information could include a summary of how many genetic variants have been discovered and possibly what is the range of their observed effect sizes.

Second, the introduction should inform what alternative models for risk prediction are available or have been investigated for the outcome of interest, including models that are based on fewer genetic variants, the same variants, non-genetic risk factors or a combination of genetic and non-genetic factors. The assessment of the performance of these risk models can provide a reference value for the evaluation of the risk model under study [13,41]. A comparison with earlier studies is most informative when essential information about the comparability of the studies is provided. Such information may include details about the setting (see below) and the age, sex and ethnicity of the population investigated.

For some topics, summarizing this information systematically would require formal systematic reviews of extensive bodies of literature and hundreds of pages, far beyond the typical short introduction of most research papers. Therefore, we recommend that the authors should be concise in reviewing the status of current risk research on the topic of interest and how the current study proposes to build on this existing evidence.

*Item 3: Specify the study objectives and state the specific model(s) that is/are investigated. State if the study concerns the development of the model(s), the validation effort of the model(s), or both.*

**Examples.** “We examined subjects in two large Scandinavian prospective studies with a median follow-up period of 23.5 years to determine whether these genetic variants alone or in combination with clinical risk factors might predict the future development of type 2 diabetes and whether these variants were associated with changes in insulin secretion or action over time.” [33]

“The present study was designed to evaluate whether the findings of Zheng et al. could be replicated in a population-based sample of American Caucasian men and to evaluate how the combination of SNP genotypes and family history function in prediction models for prostate cancer risk and for prostate cancer-specific mortality.” [31]

**Explanation.** Objectives refer to the specific research questions that are investigated in the study. For genetic risk prediction studies, the objectives should specify which models are investigated for the prediction of which outcome in which population and setting. Furthermore, authors should state whether the report concerns the development of a novel risk model (and if so, whether some sort of internal or external validation is performed) or about a replication or validation of an earlier model. Finally, any planned subgroup and interaction analyses should be specified, including *a priori* hypotheses or a statement that subgroup and interaction effects were explored without any hypothesis.

METHODS

*Item 4:* *Specify the key elements of the study design and describe the setting, locations and relevant dates, including periods of recruitment, follow-up and data collection.*

**Examples.** “The Rotterdam Study is a prospective, population-based, cohort study among 7,983 inhabitants of a Rotterdam suburb, designed to investigate determinants of chronic diseases. Participants were aged 55 years and older. Baseline examinations took place from 1990 until 1993. Follow-up examinations were performed in 1993–1994, 1997–1999, and 2002–2004. Between these exams, continuous surveillance on major disease outcomes was conducted. Information on vital status was obtained from

municipal health authorities.” [42]

“A cohort of 2,576 men and 2,636 women from a general population (aged 30–65 years at inclusion) participated in the DESIR longitudinal study and were clinically and biologically evaluated at inclusion, at 3-, 6-, and 9-year visits.” [43]

**Explanation.** Key elements about the study design include whether the analyses were performed in: a cohort study, which follows a group of individuals over time to identify incident cases of disease; a cross sectional study, which examines prevalent disease in a defined population; or a case-control study, which compares individuals with the trait of interest to those without [17,29,44]. Setting refers to how participants were recruited, for example through hospitals, outpatient clinics, screening centers or registries, and location refers to the country, region and cities, if relevant. Stating the dates of data-collection rather than the duration of the follow-up helps to place the study in historical context and is particularly important in the context of changes in diagnostic methods (e.g., imaging and use of biomarkers), and changes in the assessment of genotype and other risk factors.

Researchers should also state whether the data were *de novo* collected specifically for the purpose stated in the introduction, or whether the analyses were conducted using previously collected data [29]. The secondary use of existing data is not necessarily less credible, but a statement might help to explain limitations in the study, including, but not limited to, relevant data not being assessed or the presence of peculiar population characteristics.

*Item 5: Describe eligibility criteria for participants, and sources and methods of selection of participants.*

**Examples.** (Eligibility criteria) “The diagnosis of diabetes in case subjects was based on either current treatment with diabetes-specific medication or laboratory evidence of hyperglycemia if treated with diet alone. Patients with confirmed diagnosis of monogenic diabetes and those treated with regular insulin therapy within 1 year of diagnosis were excluded. Case subjects in this study had an age at diagnosis between 35 and 70 years, inclusive. Control subjects had not been diagnosed with diabetes at the time of recruitment or subsequently and were excluded if there was evidence of hyperglycemia during recruitment (fasting glucose >7.0 mmol/l, A1C >6.4%) or if they were >80 years old.” [45]

(Sources and methods of selection) “The study population consisted of 283 women with previous gestational diabetes mellitus who were admitted to the Department of Obstetrics, Copenhagen University Hospital, Rigshospitalet, Denmark, during 1978–1996 and who had participated in a follow-up study during 2000–2002.” [32]

**Explanation.** The predictive performance of a risk model might vary with the population in which the test is applied, and is preferably assessed by testing a random sample of individuals from the population at risk of the disease or outcome. The eligibility criteria, source and methods of selection of the study participants thus inform readers about the assumed target population for testing as well as about the representativeness of the study population. Knowledge of the selection criteria is essential in appraising the validity and generalizability of the study results. Eligibility criteria may be presented as inclusion and exclusion criteria, specifying characteristics such as age, sex, ancestry, ethnicity and/or geographical region, and, for case-control studies, diagnosis and comorbidity. The source refers to the populations from which the participants were selected and to the methods of selection—whether participants were, for example, randomly invited, referred or self-selected. The diagnostic criteria should be clearly described, including references to standards, if applicable.

For cohort and cross-sectional studies, the population base from which participants were invited (e.g., from a general population, specific region or hospital) should be specified. Depending on the aim of the cohort, typical eligibility criteria may include age, sex, ethnicity, specific risk factors, and for cohorts of patients, diagnosis, disease duration or stage, and comorbidity [29].

For case-control studies, one should specify the (diagnostic) criteria that were used to select cases, and the criteria for selecting the controls. The extent to which controls were screened for absence of symptoms related to the disease or outcome under study should be described. Description of the criteria should enable understanding of the spectrum of disease involved. Case-control studies sometimes compare very severe cases with very healthy controls, particularly if the data were previously collected primarily for gene discovery [8,46]. Such stringent selection of participants is an effective strategy for gene discovery, but predictive performance might be overestimated compared with assessment in unselected populations where controls might have early symptoms or risk factors of disease. Furthermore, for case-control studies, it is important to specify whether cases and controls were matched and how, as overmatching might affect the predictive power of that factor in the sample relative to its predictive power in an unmatched population.

*Item 6: Clearly define all participant characteristics, risk factors and outcomes. Clearly define genetic variants using a widely-used nomenclature system.*

**Examples.** (Predictors) “We selected six SNPs from six loci on the basis of their association with levels of LDL or HDL cholesterol in at least one previous study. These six SNPs were, for association with LDL cholesterol, *APOB* (apolipoprotein B, rs693), *PCSK9* (proprotein convertase subtilisin/kexin type 9, rs11591147), and *LDLR* (low-density lipoprotein receptor, rs688); and for association with HDL cholesterol, *CETP* (cholesteryl ester transfer protein, rs1800775), *LIPC* (hepatic lipase, rs1800588), and *LPL* (lipoprotein lipase, rs328).” [47]

(Predictors) Another example is provision of the information in tabular form (See Table 3) [48].

(Predictors) “We defined a positive self reported family history of diabetes as a report that one or both parents had diabetes; this definition is more than 56% sensitive and 97% specific for confirmed parental diabetes. […] We considered diabetes to be present in a parent when medication was prescribed to control the diabetes or when the casual plasma glucose level was 11.1 mmol per liter or higher or 200.0 mg per deciliter or higher at any examination.” [48]

(Outcomes) “The prespecified composite end point of cardiovascular events was defined as myocardial infarction, ischemic stroke, and death from coronary heart disease. Myocardial infarction was defined on the basis of codes 410 and I21 in the International Classification of Diseases, 9th Revision and 10th Revision (ICD-9 and ICD-10), respectively. Ischemic stroke was defined on the basis of codes 434 or 436 (ICD-9) and I63 or I64 (ICD-10).” [47]

**Explanation.** All participant characteristics, genetic and non-genetic risk factors, and outcomes that are considered and used in the analyses, should be defined and described unambiguously. Disease outcomes should be defined by reference to established diagnostic criteria or justification of study-specific criteria, if such are employed. Both the selection of genetic and non-genetic risk factors should be clarified. Authors should specify whether all known risk factors are included, and, if not, why some are excluded. Genetic variants should be described using widely-used nomenclature [49]. For example, SNPs could be presented with rs numbers with allusion to the pertinent reference database and build (e.g., HapMap release 27) [50]. When proxies (surrogate markers) are considered, the correlation with the intended variant should be quantified, for example in terms of R^2^ along with the population used to derive the correlation. When variants are obtained by imputation, the imputation method and reference database should be described along with an estimate of the quality of the imputation.

*Item 7:* *(a) Describe sources of data and details of methods of assessment (measurement) for each variable. (b) Give a detailed description of genotyping and other laboratory methods.*

**Examples.** (Sources of data) “Phenotyping was performed by the participating

gastroenterologist from each university medical center by reviewing a patient’s chart retrospectively.” [7]

(Sources of data) “All clinical measurements were performed in practice by [the first author] (first measurement) and a nurse practitioner (second, third and fourth measurements with in-between periods of 3 months).” [51]

(Methods of assessment) “Weight was measured in underwear to the nearest 0.1 kg on Soehnle electronic scales. We measured height in bare feet to the nearest 1 mm by using a stadiometer with the participant standing erect with head in the Frankfort plane. We calculated body mass index as weight (kilograms)/height (metres) squared. We measured waist circumference, taken as the smallest circumference at or below the costal margin, with participants unclothed in the standing position by using a fibreglass tape measure at 600 g tension. We measured systolic blood pressure and diastolic blood pressure twice in the sitting position after five minutes’ rest with the Hawksley random zero sphygmomanometer. We took the average of the two readings to be the measured blood pressure. We took venous blood in the fasting state or at least five hours after a light, fat free breakfast, before a two hour 75 g oral glucose tolerance test was done. Serum for lipid analyses was refrigerated at −4°C and assayed within 72 hours. We used a Cobas Fara centrifugal analyzer (Roche Diagnostics System, Nutley, NJ) to measure cholesterol and triglyceride concentrations. We measured high density lipoprotein cholesterol by precipitating non-high density lipoprotein cholesterol with dextran sulfate-magnesium chloride with the use of a centrifuge and measuring cholesterol in the supernatant fluid. We used the Friedewald formula to calculate low density lipoprotein cholesterol concentration.” [52]

(Outcomes) “Women with gestational diabetes mellitus in the years 1978–1985 were diagnosed by a 3h, 50g oral glucose tolerance test (OGTT), whereas women with gestational diabetes mellitus in 1987–1996 were diagnosed by a 3h, 75g OGTT.” [32]

(Genotyping) “Genotyping was performed with the use of matrix-assisted laser desorption–ionization time of-flight mass spectrometry on a MassARRAY platform (Sequenom), as described previously. All SNPs were in Hardy–Weinberg equilibrium (P>0.001). The genotyping success rate was 96%. Using 15 samples analyzed in quadruplicate, we found the genotyping error rate to be <0.7%.” [47]

**Explanation.** Apart from the selection and definitions of the variables, the sources and methods used for the assessment can impact the quality of the study. Important quality concerns are the potential for misclassification of risk factors and outcomes, as well as the accuracy of genotyping [29]. Sources of data basically refer to who did the data collection and how. Were the data collected by research physicians or trained students? Were questionnaires completed in an interview or based on self-report, and was the genotyping performed in house or by a specialized laboratory? Methods of assessment refer to the specific techniques or questionnaires that were used. If methods have been published previously, provide a reference. The laboratory procedures used to measure biomarkers should be described in sufficient detail for others to be able to perform them and evaluate the generalizability of prediction models that include them. For less widely-used assessments, such as questionnaires and procedures that are developed by the researchers themselves, authors should report validity and reliability information about the quality of the assessment [53]. When different assessments are used at baseline and follow-up (e.g., baseline assessments done by research physicians and follow-up assessments obtained from medical records of the general practitioner) these should be explained. When there is an arbitration process for outcomes (e.g., centralized team arbitrating on outcomes based on information contributed by local investigators in peripheral centers), this process should be specified.

*Item 8:* *(a) Describe how genetic variants were handled in the analyses. (b) Explain how other quantitative variables were handled in the analyses. If applicable, describe which groupings were chosen, and why.*

**Examples.** (Genetic variants) “Using these 18 SNPs, we constructed a genotype score ranging from 0 to 36 on the basis of the number of risk alleles [see Table 3 for coding of the risk alleles].” [48]

(Genetic variants) “For the first analysis of the effects of the polymorphic DNA variants, we used additive genetic models. In addition, we tested dominant and recessive alternative models for the best fit […]. Multivariate linear regression analyses were used to test correlations between genotype and phenotype. Non-normally distributed variables were logtransformed before analysis. The effect size of a genetic or clinical risk factor on the risk of type 2 diabetes was calculated from multivariate regression analysis, with adjustment for age and sex, with the use of Nagelkerke R square. We estimated the predictive value of a combination of risk alleles (each person could have 0, 1, or 2 of them, for a total of 22) in 11 genes, which significantly predicted the risk of diabetes by defining subjects with more than 12 risk alleles (about 20%) as being at high risk and those with fewer than 8 risk alleles (about 20%) as being at low risk.” [33]

(Other variables) “Multivariate unconditional logistic regression analysis was performed to evaluate the relationships between prevalence or progression of AMD and all the genotypes plus various risk factors, controlling for age (70 years or older versus younger than 70), sex, and education (high school or less versus more than high school), cigarette smoking (never, past, or current), and body mass index (BMI), which was calculated as the weight in kilograms divided by the square of the height in meters (<25, 25–29.9, and 30+).” [6]

**Explanation.** There are many approaches to data analysis of genetic variants; thus, specification and clarification of this handling is particularly relevant. Genetic variants may be entered in regression analysis separately as dominant or recessive effects e.g., [54,55], per allele (additive or log-additive) effects [32], or genotype categories [42,56]. Any of these three approaches can be followed depending on what was the best fitting genetic model for each variant [6–8]. Alternatively, genetic variants may be entered combined as risk scores [33,47,52]. Risk scores often simply sum the number of risk alleles or genotypes (unweighted), or sum their beta-coefficients from regression analyses (weighted). When using risk scores, authors should explain which of the alleles or genotypes is considered as the risk variant, as this is not necessarily the less common (minor) variant (see Table 3). The description of the coding of the genetic variants should enable other researchers to replicate the analyses for validation or updating of the risk model.

Quantitative variables can be handled as continuous or be categorized. Transformations may be required when the relationships between the variables and the outcome are not linear, and these should be specified. Frequently, quantitative variables are categorized before inclusion in the analyses. A well-known example is body mass index, which is categorized as underweight, normal weight, overweight and obese. The rationale and thresholds used for categorization should be explained, particularly when they deviate from commonly used cut-offs based on clinical or epidemiological studies.

*Item 9:* *Specify the procedure and data used for the derivation of the risk model. Specify which candidate variables were initially examined or considered for inclusion in models. Include details of any variable selection procedures and other model-building issues. Specify the horizon of risk prediction (e.g., 5-year risk).*

**Examples.** (Model derivation) “We constructed multivariable proportional-hazards models to examine the association between the genotype score and the time to the first cardiovascular event, excluding subjects who had had a previous myocardial infarction or ischemic stroke. We first confirmed that the proportional-hazards assumption was met. The hazard ratio for the genotype score as a continuous measure was estimated in a model adjusting for all 14 available baseline covariates. Cumulative incidence curves were constructed according to the genotype score with the use of Cox regression analysis.” [47]

(Variable selection) “Twenty-three candidate genes involved in the pathogenesis of inflammation and myocardial ischemia-reperfusion injury were selected a priori based on previous transcription profiling in humans and animal models, pathway analysis, a review of linkage and association studies reported in the literature, and expert opinion. Forty-eight SNPs were subsequently selected in these process-specific candidate genes, based on literature review, genomic context, and predictive analyses with an emphasis on functionally important variants.” [54]

(Model building issues) “Both univariate and multivariate odds ratios (ORs) were calculated with a binary-logistic regression model … to evaluate the relationship between polymorphisms and prevalent CVD. For that purpose, dummy variables were created using the homozygous wild-type genotype as reference category. Age and gender, both demographic variables, were incorporated in both the univariate as well as in the multivariate linear regression analyses … Adjustment for potential confounders was performed by incorporating smoking, alcohol, diabetes mellitus, waist circumference, serum creatinine, mean systolic and diastolic blood pressure, microalbuminuria and dyslipidaemia into these models. To avoid collinearity, waist circumference was used instead of waist-to-hip ratio or body mass index and condensed measures such as diabetes and dyslipidaemia were used, as defined earlier.” [51]

**Explanation.** Because of the potential for flexibility in the derivation of the risk model, authors need to clarify why and how they constructed the model as they did and which data they used. This clarification includes a specification of the variables, defined in item 6, that were initially considered and which procedures were followed for a final selection (e.g., backward deletion or forward inclusion, and the criteria for deletion and inclusion), if applicable. Clarification also includes a specification of the study participants included in the analysis, if different from the total study population, transformations of the variables, the choice of statistical model (e.g., logistic or Cox proportional hazards models), and the handling of interaction effects between predictors in the model (see also item 13). The specification also concerns the rationale for constructing separate models for subgroups, e.g., for different ethnic groups, or including the stratification variable as a variable or interaction effect in a model for the total population.

Authors should also specify and explain the horizon of the risk prediction, when appropriate (e.g., in cohort studies, whether the model predicts, for instance, 5-year or lifetime risk). When more complicated risk prediction models are developed using statistical learning methods such as regularized regression or support vector machines, these should be explained and specified in sufficient detail that others can implement these models in other data sets. For some more complex “black box” models (such as random forests) this may require making a software implementation of the final model available. The description of the data used should include whether a selection of the population was used for the derivation of the model, how this subpopulation was selected, and how censored data were handled in cohort studies.

Some studies aim only to validate and further apply an already existing model. In this case, it should simply be stated that a previous model was used with appropriate reference to the previous study or studies that developed the model along with a succinct description of its features.

*Item 10:* *Specify the procedure and data used for the validation of the risk model.*

**Example.** “The internal validity of the prediction models was assessed using bootstrapping techniques. A total of 100 random bootstrap samples were drawn with replacement from the [total] group of 1,337 patients. The discriminative accuracy of the 100 prediction models as fit on these bootstrap samples was determined for each bootstrap sample and for the original group (n =1,337). This comparison gives an impression of how “overoptimistic” the model is, i.e., how much the performance of the model would deteriorate when applied to a new group of similar patients.” [36]

“Evaluation of model predictive performance using the same dataset used for fitting the model usually leads to a biased assessment. To obtain an unbiased assessment of discriminatory power of the multivariate regression models, a tenfold cross-validation was used in the ROC analysis and in the IDI analysis. Tenfold crossvalidation randomly divides the data into ten (roughly) equal subsets and repeatedly uses any nine subsets for model fitting and the remaining subset as validation until each of the ten subsets has been used exactly once as validation data.” [57]

**Explanation.** Assessment of the risk model in the same population as that from which the model was derived generally leads to more positive conclusions than when the evaluation is conducted in an independent population [58]. Therefore, validation of the risk model, reassessing the performance of the model in another dataset, is an essential part of model evaluation [59], especially when models are developed with the specific intention to apply them in health care. There are two main types of validation: internal validation in the same population or external validation in an independent sample. Internal validation is useful to prevent optimistic assessments, but it does not inform about the performance of the model in other samples of the same population [60]. Moreover, many methods of standard internal validation, such as cross-validation, can still give inflated estimates of classification accuracy, even if properly performed. Authors should report whether they performed (internal or external) validation, and describe the procedure of the validation process. For example, for internal validation, authors should describe what part of the population was used to derive the risk model and what part was used for the validation, and whether they, for example, used cross validation and bootstrapping techniques [60]. For external validation, they should describe the populations that are used for the validation, particularly the comparability with the population that was used to derive the risk model. If the model is already validated elsewhere in previous research, this should also be stated. So far, none of the genetic risk prediction studies had performed an external validation of the risk model [3].

*Item 11:* *Specify how missing data were handled.*

**Examples.** “Variables with missing values were hypertension (1%), smoking (10%), BMI (14%), plasma HDL cholesterol (19%), plasma LDL cholesterol (20%), and plasma triglycerides (16%). We applied a multiple imputation method (aregImpute function of the R statistical package; version 2.5.1; www.r-project.org) to impute these missing values in our Cox proportional hazards models because imputation decreases bias in the hazard ratios that may occur when patients with incomplete information are excluded from the analysis. In a secondary analysis, we used the full data set (n = 2,145) and multiple imputation to impute both missing values for conventional risk factors and missing genotype data. This analysis gave discriminative accuracies for the 3 prediction models virtually identical to the analysis without imputation of missing genotype data […].” [36]

**Explanation.** Missing data are inevitable in observational studies. Authors should specify the percentage of missing values in their data, indicate whether there are theoretical or empirical grounds that missingness could be non-random, and specify how missing data were handled in the analyses. Authors should specify the methods used to deal with the missing data (e.g., complete case analysis, imputation, reweighting) and the assumptions that underlie this choice. Assumptions may include the distribution of the data and whether data were missing completely at random, or related to other variables, including the outcome of the study [61].

*Item 12: Specify all measures used for the evaluation of the risk model including, but not limited to, measures of model fit and predictive ability.*

**Examples.** “We calculated odds ratios and 95% confidence intervals associated with each additional risk allele for each SNP individually and in the genotype score. Using C statistics …, we evaluated the discriminatory capability of the models with the genotype score as compared with the models without the genotype score. We also evaluated risk reclassification with the use of the genotype score, according to the method developed by Pencina et al. for determining net reclassification improvement. We assessed model calibration using the Hosmer–Lemeshow chi-square test. We used categories of genotype score to calculate likelihood ratios and posterior probabilities of diabetes. Statistical analyses were performed with the use of SAS software, version 8 (SAS Institute). A two-tailed P value of less than 0.05 was considered to indicate statistical significance.” [48]

“Our primary measure of discrimination was the Harrell c-index, a generalization of the area under the receiver-operating characteristic curve that allows for censored data. The c-index assesses the ability of the risk score to rank women who develop incident cardiovascular disease higher than women who do not. We assessed general calibration across deciles of predicted risk by using the Hosmer–Lemeshow goodness-of-fit test to compare the average predicted risk with the Kaplan–Meier risk estimate within each decile and considered a chi-square value of 20 or higher (P < 0.01) to be poor calibration. We assessed risk reclassification by sorting the predicted 10-year risk for each model into 4 categories (<5%, 5% to <10%, 10% to <20%, and ≥20%). We then compared the assigned categories for a pair of models. For each pair, we calculated the proportion of participants who were reclassified by the comparison model versus the reference model; we considered reclassification to be correct if the Kaplan–Meier risk estimate for the reclassified group was closer to the comparison category than the reference. We computed the Hosmer–Lemeshow statistic for the reclassification tables, which assesses agreement between the Kaplan–Meier risk estimate and predicted risk within the reclassified categories. We also computed the Net Reclassification Improvement, which compares the shifts in reclassified categories by observed outcome, and the Integrated Discrimination Improvement, which directly compares the average difference in predicted risk for women who go on to develop cardiovascular disease with women who do not for the 2 models, on the women who were not censored before 8 years.” [56]

**Explanation.** A thorough assessment of a risk prediction model comprises many different aspects, but generally includes at least the following questions: (1) How well does the model fit the underlying data?; and (2) What is the predictive ability of the model? Several measures are available to answer each question, and the methods section should clearly describe which measures were used to answer which questions [4,62]. Measures of model fit (also referred to as calibration) include the Hosmer Lemeshow statistic, R^2^, log-likelihood and Akaike information criterion (AIC), and measures of predictive ability (also called discrimination measures) include the area under the receiver operating characteristic curve (AUC), discrimination slope and Brier score. These measures can be accompanied by figures and tables, including calibration plots (see in [60]), risk distributions (see Figure 1), AUC plots (see Figure 2), discrimination plots (see in [63]) and predictiveness curves (see in [64]). The description of the methods used should clarify also what measures of uncertainty are employed (e.g., 95% confidence intervals) and specify any tests used to determine the significance of the findings. When p-values are reported, authors should indicate what p-value threshold they considered for statistical significance.

When two risk models are compared and one is an expanded version of the other, the assessment of the risk models includes the two questions for each model. Increases in AUC or in discrimination slope (called integrated discrimination improvement, IDI) provide simple ways to assess improvement of one model over the other [58]. Recent studies have also assessed whether the improvement of risk models also reclassifies people into different risk categories [2,65]. These measures of reclassification, such as the percentage of total reclassification and net reclassification improvement [4,66], are calculated from a reclassification table (Table 7). When risk categories are used (e.g., for the calculation of reclassification measures), the rationale for the cut-off values should be presented with either appropriate reference to previous work showing that this is a standard choice, or appropriate justification for the choice of cut-offs made by the authors. When several different cut-off categorizations have been studied, all of them should be reported.

*Item 13:* *Describe all subgroups, interactions and exploratory analyses that were examined*

**Examples.** (Subgroups) “[In introduction:] However, it remains unknown whether all these genetic and environmental factors act independently or jointly and to what extent they as a group can predict the occurrence of AMD or progression to advanced AMD from early and intermediate stages. Such information may be useful for screening those at high risk due to a positive family history or having signs of early or intermediate disease, among whom some progress to advanced stages of AMD with visual loss. … [In Methods:] Individuals with advanced AMD were compared to the control group of persons with no AMD, and progressors were compared to nonprogressors

with regard to genotype and risk factor data.” [6]

(Interactions) “Multiplicative interactions were tested for each pair of [all 6] SNPs by including both main effects and an interaction term (a product of two main effects) in a logistic regression model.” [67]

**Explanation.** For the evaluation of the predictive performance there might be subgroups in which the risk model performs better than in the initial study population, and there might be genetic variants that jointly have a larger impact on disease risk. The large number of possible analyses that include subgroups or interactions, however, increases the likelihood of finding at least some statistically significant effect by chance [68]. Authors should therefore not only clarify all additional subgroup analyses they performed, but also indicate whether these were planned based on *a priori* clinical or epidemiological evidence, or arose in an exploratory fashion. Similarly, authors should also explain whether interaction effects were considered and, if so, which ones and why, and how the selection in the final model was done (see item 9). These descriptions should include any methods used to prevent over interpretation of the results, e.g., methods that adjust the p-value thresholds to adjust for multiple testing. Planned analyses of subgroups and interactions should logically follow from the introduction (see item 3); exploratory analyses can be introduced in the methods.

***RESULTS***

*Item 14:* *Report the numbers of individuals at each stage of the study. Give reasons for non-participation at each stage. Report the number of participants not genotyped, and reasons why they were not genotyped.*

**Examples.** “Among 3648 identified subjects with prostate cancer, 3161 (87%) agreed to participate. DNA samples from blood, tumor–node–metastasis (TNM) stage, Gleason grade (as determined by biopsy), and levels of prostate-specific antigen (PSA) at diagnosis were available for 2893 subjects (92%).” [67]

“[In methods:] In short, the Rotterdam Study is a prospective, population based, cohort study among 7,983 inhabitants of a Rotterdam suburb, designed to investigate determinants of chronic diseases … [In Results:] A total of 6,544 participants were successfully genotyped for at least one polymorphism. Complete genotype information on all polymorphisms was present in 5,297 subjects (of whom 490 were incident cases and 545 were prevalent cases).” [42]

**Explanation.** The study report should clearly present the number of participants that were eligible for the study and how many were included in the final analyses. The authors should report the main reasons for non-participation, so that the reader can judge the extent to which the population available for the analyses is a representative selection of those who were eligible. Any evidence for missingness not completely at random should be presented [69]. A flowchart can help clarify complex datasets, and is particularly useful for follow-up studies. A flowchart presents the exact numbers and the structure of the study (see example in [29]). When a flowchart of the study has been previously published and the flow of participants is the same, a reference to the earlier publication can save space. For cohort studies, descriptive information about the follow-up time, e.g., in terms of the range, median and interquartile range of follow-up duration, should be provided.

Frequently, studies do not have complete genotype information for all participants for many reasons, including budget issues, unavailability of DNA material and genotyping quality issues. Because some reasons might impact the validity of the study, the number of participants that were not genotyped and the reasons should be reported. An example is survivor bias, which might occur when genotyping is performed on DNA obtained in one of the follow-up assessments of a cohort study (see example [52]).

*Item 15:* *Report demographic and clinical characteristics of the study population, including risk factors used in the risk modeling.*

**Examples.** “The mean age of cases was similar to that of controls, 59.9 and 59.6 years, respectively. In comparison with controls, a higher proportion of cases had a first-degree family history of prostate cancer (see Table 4). The majority of cases had serum PSA values of 4.0–9.9 ng/ml at diagnosis, localized stage disease and Gleason

scores of 5 or 6; most were treated with radical prostatectomy.” [31]

**Explanation.** The authors should describe their populations in as much detail as is needed for the readers to judge the generalizability of the results. This description should include relevant demographic information, such as age, sex and ethnicity, and information on other risk factors and relevant pathology, e.g., early disease characteristics and comorbidity. Continuous variables are preferably described by means and standard deviations, and when their distributions are skewed, by medians and inter-quartile ranges. Variables that have a small number of response categories are preferably presented as percentages and numbers. This descriptive information is preferably presented separately for those people with and without the outcome of interest.

*Item 16:* *Report unadjusted associations between the variables in the risk model(s) and the outcome. Report adjusted estimates and their precision from the full risk model(s) for each variable.*

**Examples.** “Table 5 displays the unadjusted association between demographic, environmental, and genetic variables and incident advanced AMD as well as the sample sizes within the groups. All factors except gender were related to progression. Baseline macular status was strongly related to progression. Both modifiable factors (smoking and BMI) and genetic variants were also associated with worsening of macular disease over time. The antioxidant/mineral treatment group had a lower rate of progression. … Table 6 displays the multivariate adjusted ORs for incident advanced AMD and shows that, after adjustment for genotypes, older age, smoking, and higher BMI were related to a higher rate of progression. Baseline grade was a strong predictor of incident advanced AMD, and antioxidant–mineral treatment was protective. The two CFH variants each independently increased risk of progression about two- to threefold, with similar increased risk for C3, comparing the homozygous risk and nonrisk genotypes. Variants in the two complement genes C2 and CFB reduced risk, although the association with CFB was not significant for progression to incident advanced AMD. …” [6]

**Explanation.** To understand which risk factors have contributed to the distribution in risk predictions, authors should report model estimates for each, e.g., regression coefficients, such as odds ratios or hazard ratios, and confidence intervals from each full model considered for all risk factors included. Adjusted estimates should be presented next to the unadjusted estimates, so that readers are able to judge the extent to which the findings change by the inclusion of other risk factors in the model. This is particularly relevant for models that combine genetic and non-genetic risk factors, because non-genetic risk factors can be intermediate factors in the biological pathway [41] and many non-genetic risk factors have complex correlation patterns [70,71]. Note that several studies have presented adjusted effect sizes for genetic variants (e.g., [42,48,51]) that were adjusted only for non-genetic risk factors. This is not the same as effect sizes for genetic variants from the full model, where coefficients are additionally adjusted for the other genetic variants as well. When regression methods were used for the prediction of risks, the intercept of the full model should be reported to facilitate future replication and validation of the risk model (see Table 6). For complex models where exhaustive specification of parameter estimates is not feasible, authors should provide software implementations of the risk prediction algorithm (see item 24).

*Item 17:* *Report distributions of predicted risks and/or risk scores.*

**Examples.** The distribution of predicted risks or risk scores is best presented in a figure, see Figure 1 [45].

**Explanation.** Distributions of predicted risks inform the reader about the spread of risks in the population, as well as the frequencies at the higher and lower ends of the distribution. Preferably the report should present separate distributions for participants with and those without the outcome of interest, as this illustrates the discriminative accuracy of the risk model. The more the two distributions disperse, the higher the AUC. Authors should label the highest and lowest category by their actual range at least once. For example, Figure 1 shows that the lowest category is labeled 10-11 risk alleles, rather than 0-11, which informs readers that none of the participants had 0 to 9 risk alleles.

*Item 18:* *Report measures of model fit and predictive ability, and any other performance measures, if pertinent.*

**Examples**. “We also evaluated whether genetic risk factors would further increase the risk imposed by an increase in the BMI or a decrease in the disposition index. There was a stepwise increase in diabetes risk with an increasing number of risk alleles and increasing quartiles of BMI (Fig. [not shown]) or a disposition index above or below the median. Therefore, carriers of more than 12 risk alleles who were in the highest quartile of BMI (263 of 826 subjects vs. 45 of 874 subjects) or who had a low disposition index (58 of 153 subjects vs. 17 of 168 subjects) had an odds ratio for type 2 diabetes of 8.0 (95% CI, 5.71 to 11.19; P = 9.1×10^−34^) and 5.8 (95% CI, 3.18 to 10.61, P = 1.1×10^−8^), respectively (Fig. [not shown]). The C statistics had minimal yet significant improvement after the addition of data from the genotyped DNA variants to the clinical model (from 0.74 to 0.75, P = 1.0×10^−4^) (Supplementary Table [not shown]). … we also reclassified subjects into three risk categories (0 to ≤10%, >10 to ≤20%, and >20%) using the net-reclassification-improvement method (Supplementary Table [not shown]). By adding genetic factors to clinical factors, we could reclassify 9% of the MPP subjects (P = 2.5×10^−5^) and 20% of the Botnia subjects (P = 0.05) to a higher risk category. Also, the use of the integrated-discrimination- improvement method, which did not require predefined risk categories, significantly improved the prediction of future diabetes in both the MPP subjects (P = 3.7×10^−14^) and the Botnia subjects (P = 0.001).” [33]

**Explanation.** All measures of model performance that are reported in the Results section should be described in the Methods section (see item 12), so that it is clear which measure is assessed to answer which research question. As described in item 12, assessment of the performance of the genetic risk prediction model should include at least measures of model fit and predictive ability. For measures of interest, confidence intervals or other pertinent measures of uncertainty for the estimated values should be reported, wherever appropriate.

*Item 19:* *Report any validation of the risk model(s).*

**Examples.** (Internal validation) “The epidemiologic-genetic model fitted our data well with AUC of 0.80 (95% CI, 0.77 to 0.82), 0.81 (95% CI, 0.77 to 0.85), and 0.80 (95% CI, 0.76 to 0.83) for the combined, training, and validation data sets, respectively (Table [not shown]). … The leave one out validation algorithm yielded an average prediction error rate of 28.0%, 27.8%, and 27.9% for patient cases, controls, and all samples, indicating relatively high discriminatory prediction accuracy of the model.” [37]

(External validation) “We used independent GWAS samples to replicate the polygenic component, to examine whether this component is shared with bipolar disorder, and to demonstrate specificity by considering non-psychiatric diseases. We used the entire International Schizophrenia Consortium (ISC) for the discovery sample […]. The ISC-derived score was highly associated with disease in both European schizophrenia samples (Fig [not shown]).” [72]

**Explanation.** Essentially, the measures that need to be presented for the validation analyses are the same as reported for the assessment of the performance of the model in the derivation population (see items 12 and 18). If, for reasons of space, authors have to choose between presenting detailed assessment for the derivation or validation data, they should choose to report the validation analyses in more detail.

*Item 20: Present results of any subgroup, interaction or exploratory analyses, whenever pertinent.*

**Examples.** “Finally we estimated the two-way interaction between each combination of the 19 variants (171 combinations) (Supplementary Table [not shown]) and the result demonstrated few, probably spurious, associations (p<0.05). As none of the associations was significant after Bonferroni correction we believe that an additive model between each variant is acceptable. Additionally, we calculated the AUC under an ROC curve in which a model including all variants (additive) is compared with a model including a two-way interaction term in addition to the variants (interaction). The results showed that if interaction is included an AUC of 0.56 is reached, which indicates reduced discriminatory value (Supplementary Table [not shown]).” [30]

“The predictive accuracy of these 4-gene genotypes was not significantly different in study participants in the lowest, middle, or top tertile of conventional risk factor score [0.65 (0.55– 0.75), 0.63 (0.55– 0.72), and 0.60 (0.53– 0.67), respectively; P=0.66]. Adding *APOE* genotype alone significantly improved the AUC [0.68 (0.64–0.72); P<0.01 vs conventional risk factors only], but none of the other genotypes singly or in pairwise combinations did so (see Table [not shown]).”[55]

**Explanation.** In the presentation of subgroups it should be clear which findings follow from pre-specified hypotheses and which follow from exploration of the data. This distinction is particularly important for the discussion, as exploratory analyses might lead to incidental findings that need more cautious interpretation and replication [73].

**DISCUSSION**

*Item 21: Discuss limitations and assumptions of the study, particularly those concerning study design, selection of participants, measurements and analyses, and discuss their impact on the results of the study.*

**Examples.** “One of the limitations of our study is that the 18 SNPs we included are probably insufficient to account for the familial risk of diabetes. They account for a minority of diabetes heritability, and the SNP array platforms from which they were chosen capture only approximately 80% of common variants in Europeans. …

Our study has other limitations. There were few significant associations between individual risk alleles and diabetes in the Framingham Offspring Study cohort, but this finding was expected, given that alleles of small effect were tested in a community-based sample of modest size, and the aggregate set of 18 SNPs was predictive of new cases of diabetes. The participants in the Framingham Offspring Study are essentially all of European ancestry; allelic variation may require that different SNPs be used to generate a genotype score in different ancestry groups. Our genotype score gave all alleles the same weight; this may not be a true reflection of the biologic basis of type 2 diabetes. We considered the marginal value of the genotype score after accounting for only phenotypic risk factors, without consideration of behavioral risk factors for diabetes. We expect that accounting for unhealthful behaviors associated with the risk of diabetes would only further diminish the discriminatory capacity of a genotype score. However, persons with relatively less healthful lifestyle behaviors might be more susceptible to genetic risk than those with more healthful behaviors. Whether the genotype score would have value in predicting the risk of diabetes in specific subgroups that have an elevated risk on the basis of poor health habits remains to be tested.” [48]

**Explanation.** The interpretation of the study should take proper account of the results in light of all analyses that were performed. Caution in the interpretation is warranted when there was considerable opportunity for flexibility in the analyses, e.g., when arbitrary categories of predicted risks were considered or when many subgroup analyses were done. Most studies avoid a comprehensive discussion of limitations [74] and many authors admit that they fear that discussion of limitations might make their paper less attractive for publication [75]. However, this is not the case [76]. A discussion of the limitations of the study should help the reader in interpreting the validity of the findings. The description of the limitations should include not only the sources of potential bias and confounding that might have affected the results, but also the direction and magnitude of their effect [29]. An informative discussion addresses issues in the design and analyses of the study that might lead to alternative interpretations of the data than the one presented in the paper. These can refer to issues that directly influence the results, but also to issues that lead to different inferences drawn about things such as the health care relevance of the findings. Examples include characteristics of the study population, selection of participants, procedures and measures used in data collection, length of follow-up, unaccounted multiplicity of analyses, and missing data. Any possible threats to the validity of the results should be addressed in the discussion.

*Item 22:* *Give an overall interpretation of results considering objectives, limitations, multiplicity of analyses, results from similar studies, and other relevant evidence.*

**Examples.** “In this study, we show that, at a population level, accumulation of several susceptibility genes for diabetes is accompanied by a substantial increase in the risk of having the disease. This was particularly apparent, in terms of prevalence, among obese individuals. We also show that the weighted genetic score added some information that was not captured by clinical variables, including family history of diabetes. The present data also show that weighting the genetic score with the reported effect of risk alleles provided more predictive value than an unweighted genetic score generated by counting the number of risk alleles. The clinical usefulness of the score, however, remains to be demonstrated.

The present population-based cross-sectional study is in line with two very recently published prospective studies. In both of these studies, a high unweighted genetic score was associated with a marked increase in the incidence of diabetes. However, the predictive value of this score beyond clinical variables was modest.” [57]

**Explanation.** The interpretation of the study should compare the study with that of others. Other studies can include genetic prediction studies on the same outcome, but also studies that have investigated non-genetic or combined models. This discussion should compare not only the main results, but also address whether the design and conduct of the studies were comparable. Specific attention should be given to the genetic variants included in the risk models, because their number increases with the rapid developments in gene discovery. Ideally the discussion of other studies should be systematic and any relevant systematic reviews and meta-analyses might be helpful to employ in this setting [77]. It would be worthwhile discussing whether previous evidence is considered to be subject to selective reporting, which might be quite prevalent in prognostic research [10].

*Item 23:* *Discuss the generalizability and, if pertinent, the health care relevance of the study results.*

**Examples.** “Although prospective, the Whitehall II study is workplace based and therefore not necessarily representative of the general population. However, the excellent performance in Whitehall II of the non-genetic risk functions for type 2 diabetes, both of which were developed and validated in general populations, suggests that this is unlikely to bias our conclusions substantially. Moreover, our findings are consistent with those of prospective studies set in representative general populations. Our findings are also not generalizable to people of non-European ancestry, who we excluded from this analysis. Although DNA was collected some time after baseline, which could have introduced a survivor bias, we think that this is unlikely to have affected our results given the modest effect of the alleles we studied on risk of diabetes and the long natural history of the development of the life threatening complications of diabetes ... Phenotype based risk models (the Framingham offspring and Cambridge risk scores) provided greater discrimination for type 2 diabetes than did models based on 20 common independently inherited alleles associated with risk of type 2 diabetes. The addition of 20 common genotypes associated with modest risk to phenotype based risk models produced only minimal improvement in the accuracy of risk estimation assessed by recalibration and at best a minor net reclassification improvement. The major translational application of the currently known common, small effect genetic variants influencing susceptibility to type 2 diabetes is likely to come from the insight they provide on causes of disease and potential therapeutic targets.” [52]

**Explanation.** Generalizability refers to the external validity or applicability of the risk model in other populations than the one used for the development of the model. Discussion of generalizability should include reference to the representativeness of the study population in comparison with the (future) target population for testing. Differences in key demographic variables, such as in sex, age and important risk factors, should be mentioned.

While most studies currently do not have direct relevance to health care or disease prevention [41], genetic risk prediction studies sometimes are interpreted with too much optimism [60,78] and expectations run high [79]. One of the reasons is that clinical or public health relevance is concluded from statistical significance. However, in large-scale population-based studies, minor increases in predictive performance or low percentages of reclassification could be statistically significant without being clinically relevant. When interpreting the clinical relevance, authors should consider 1) the efforts it takes to obtain the additional genotype information, 2) the impact genetic results might have on medical or public health decision making and on expected health benefits, and finally, 3) the extent to which these benefits will outweigh the potential harms related to genetic testing and be affordable. A consideration of what information is needed might help prevent overoptimistic interpretations. Describe what evidence is still needed before health care implementation can be considered, for example by referencing the stage of translational research the study fits in [1]. The latter also includes a reflection on whether the population investigated is an appropriate representation of the target population and whether the risk period (e.g., 5-year risk) and the outcome are clinically relevant.

***SUPPLEMENTARY INFORMATION***

*Item 24: State whether databases for the analyzed data, risk models and/or protocols are or will become publicly available and if so, how they can be accessed.*

**Explanation.** With the advances in genomics research, data and analytic plans of genetic risk prediction studies become increasingly complex. So far, most empirical studies have used logistic or Cox proportional hazards regression analyses [3], but other methods including support vector machine learning, fuzzy logic, neural networks and classification trees have been applied [80,81]. There is increasing appreciation that it is important for these complex and extensive data to be publicly available. This allows scrutiny of the process and the results by other investigators, and appropriate use of these data for further analyses, e.g., validation studies and meta-analyses. For some ‘omics’ fields, public availability of data and protocols is a prerequisite for publication in specific journals, e.g., all gene expression profiling studies need to do this as a prerequisite for publication in *Nature Genetics* (http://www.nature.com/authors/editorial_policies/availability.html), although full deposition of data and protocols has not yet been achieved [82]. For studies of gene-phenotype associations, initiatives such as the Database of Genotype and Phenotype (dbGAP) and the Genetic Association Information Network (GAIN) are promoting the public data availability of genotype-phenotype data [83,84].

***FUNDING***

*Item 25: Give the source of funding and the role of the funders for the present study. State whether there are any conflicts of interest.*

**Examples.** “This study was supported by grants from the National Heart, Lung, and Blood Institute and National Cancer Institute, National Institutes of Health; the Donald W. Reynolds Foundation; and the Leducq Foundation. Additional support for DNA extraction, reagents, and data analysis was provided by Roche Diagnostics and Amgen. Genotyping of the 9p21.3 variant was performed by Celera. The funding sources had no role in the design, conduct, or reporting of this study or the decision to submit the manuscript for publication.”[56]

**Explanation.** Authors should disclose any funding they received to carry out the study, and state what the role of the funding agency/agencies was in the design, conduct and analyses of the study. Given the potential commercial interests in predictive tests that could be used in large populations of diseased and healthy people, both inside and outside health care practice, any financial or other conflicts of interest should be transparent. Conflicts of interest can impact all stages of the research, including the study design, choice of exposures, outcomes, statistical methods and selective interpretation and publication of results [29,85].

**Concluding remarks and future directions**

High quality reporting reveals the strengths and weaknesses of empirical studies, facilitates the interpretation of the scientific and health care relevance of the results, in particular within the framework of systematic reviews and meta-analyses, and helps build a solid evidence base for moving genomic discoveries into applications in health care practice. The GRIPS guidelines were developed to improve the transparency, quality and completeness of the reporting of genetic risk prediction studies. GRIPS does not prescribe how studies should be designed, conducted and analyzed, and therefore, the guidelines should not be used to assess the quality of empirical studies [86]. The guidelines should only be used to check whether all essential items are adequately reported.

The GRIPS guidelines were developed by a multidisciplinary group of 25 experts, seven of whom were also part of the STREGA initiative [17]. Taking advantage of their earlier work, we organized the GRIPS workshop and manuscript writing along the same lines. The strategy we followed in developing our guidelines is consistent with the recommendations proposed in a recent paper on how to develop health research reporting guidelines [87], which was published after our workshop. In short, we had reviewed genetic risk prediction studies and identified the need for guidance [3,41], prepared a proposal for GRIPS on the basis of previous guidelines for other studies [17–19], organized a workshop to discuss each item of the proposal in-depth, had several consultation rounds for the writing of this paper and pilot-tested the checklist.

Guidelines have been developed for a wide range of empirical and review studies [15], but it should be acknowledged that their uptake and impact on reporting has not been extensively investigated. Several studies have shown that reporting guidelines do improve the reporting of studies, but there is still room for improvement [88,89]. For example, a comparison of randomized controlled trials published in 2000 and in 2006 showed that more recommended items were addressed in the papers, but that reporting remained suboptimal [89]. Fortunately, correct reporting of items was more frequent among papers published in journals that endorsed the CONSORT guidelines compared with journals that did not. We agree with the researchers from the EQUATOR project that “ [reporting guidelines] improve the accuracy and transparency of publications, thus facilitating easier and more reliable appraisal of quality and relevance” [90].

The methodology for designing and assessing genetic risk prediction models is still developing. For example, newer measures of reclassification were first introduced in 2007 [91] and several alternative reclassification measures have been proposed [4]. Which measures to apply and when to use measures of reclassification is still subject to ongoing evaluation and discussion [65]. Furthermore, alternative strategies for constructing risk models other than simple regression analyses are being explored, and these might add increasing complexity to the reporting. In formulating the items of the GRIPS Statement, these methodological advances were anticipated. It was for this reason that the GRIPS Statement recommends how a study should be reported and not how a study should be conducted or analyzed. Therefore, methodological and analytical developments will not immediately impact the validity and relevance of the items, but the GRIPS statement will be updated when this is warranted by essential new developments in the construction and evaluation of genetic risk models.

**References**

1. Khoury MJ, Gwinn M, Yoon PW, Dowling N, Moore CA et al. (2007) The continuum of translation research in genomic medicine: how can we accelerate the appropriate integration of human genome discoveries into health care and disease prevention? Genet Med 9: 665-674.

2. Hlatky MA, Greenland P, Arnett DK, Ballantyne CM, Criqui MH et al. (2009) Criteria for evaluation of novel markers of cardiovascular risk: a scientific statement from the American Heart Association. Circulation 119: 2408-2416.

3. Janssens ACJW, Van Duijn CM (2009) Genome-based prediction of common diseases: methodological considerations for future research. Genome Med 1: 20.

4. Pencina MJ, D'Agostino RB, Sr., D'Agostino RB, Jr., Vasan RS (2008) Evaluating the added predictive ability of a new marker: from area under the ROC curve to reclassification and beyond. Stat Med 27: 157-172.

5. Mihaescu R, van Zitteren M, van Hoek M, Sijbrands EJ, Uitterlinden AG et al. (2010) Improvement of risk prediction by genomic profiling: reclassification measures versus the area under the receiver operating characteristic curve. Am J Epidemiol 172: 353-361.

6. Seddon JM, Reynolds R, Maller J, Fagerness JA, Daly MJ et al. (2009) Prediction model for prevalence and incidence of advanced age-related macular degeneration based on genetic, demographic, and environmental variables. Invest Ophthalmol Vis Sci 50: 2044-2053.

7. Weersma RK, Stokkers PC, van Bodegraven AA, van Hogezand RA, Verspaget HW et al. (2009) Molecular prediction of disease risk and severity in a large Dutch Crohn's disease cohort. Gut 58: 388-395.

8. Wang J, Ban MR, Zou GY, Cao H, Lin T et al. (2008) Polygenic determinants of severe hypertriglyceridemia. Hum Mol Genet 17: 2894-2899.

9. Kyzas PA, Denaxa-Kyza D, Ioannidis JP (2007) Quality of reporting of cancer prognostic marker studies: association with reported prognostic effect. J Natl Cancer Inst 99: 236-243.

10. Kyzas PA, Loizou KT, Ioannidis JP (2005) Selective reporting biases in cancer prognostic factor studies. J Natl Cancer Inst 97: 1043-1055.

11. McShane LM, Altman DG, Sauerbrei W, Taube SE, Gion M et al. (2005) REporting recommendations for tumor MARKer prognostic studies (REMARK). Nat Clin Pract Urol 2: 416-422.

12. Kyzas PA, Denaxa-Kyza D, Ioannidis JP (2007) Almost all articles on cancer prognostic markers report statistically significant results. Eur J Cancer 43: 2559-2579.

13. Tzoulaki I, Liberopoulos G, Ioannidis JP (2009) Assessment of claims of improved prediction beyond the Framingham risk score. JAMA 302: 2345-2352.

14. von Elm E, Egger M (2004) The scandal of poor epidemiological research. BMJ 329: 868-869.

15. Simera I, Moher D, Hoey J, Schulz KF, Altman DG (2010) A catalogue of reporting guidelines for health research. Eur J Clin Invest 40: 35-53.

16. Von Elm E, Altman DG, Egger M, Pocock SJ, Gotzsche PC et al. (2007) The Strengthening the Reporting of Observational Studies in Epidemiology (STROBE) statement: guidelines for reporting observational studies. PLoS Med 4: e296.

17. Little J, Higgins JP, Ioannidis JP, Moher D, Gagnon F et al. (2009) STrengthening the REporting of Genetic Association Studies (STREGA): an extension of the STROBE statement. PLoS Med 6: e22.

18. Bossuyt PM, Reitsma JB, Bruns DE, Gatsonis CA, Glasziou PP et al. (2003) Towards complete and accurate reporting of studies of diagnostic accuracy: the STARD initiative. BMJ 326: 41-44.

19. McShane LM, Altman DG, Sauerbrei W, Taube SE, Gion M et al. (2005) Reporting recommendations for tumor marker prognostic studies. J Clin Oncol 23: 9067-9072.

20. Plint AC, Moher D, Morrison A, Schulz K, Altman DG et al. (2006) Does the CONSORT checklist improve the quality of reports of randomised controlled trials? A systematic review. Med J Aust 185: 263-267.

21. Khoury MJ, Dorman JS (1998) The Human Genome Epidemiology Network. Am J Epidemiol 148: 1-3.

22. Freedman AN, Seminara D, Gail MH, Hartge P, Colditz GA et al. (2005) Cancer risk prediction models: a workshop on development, evaluation, and application. J Natl Cancer Inst 97: 715-723.

23. Khoury MJ, Gwinn M, Ioannidis JP (2010) The emergence of translational epidemiology: from scientific discovery to population health impact. Am J Epidemiol 172: 517-524.

24. Moons KG, Altman DG, Vergouwe Y, Royston P (2009) Prognosis and prognostic research: application and impact of prognostic models in clinical practice. BMJ 338: b606.

25. Von Elm E, Altman DG, Egger M, Pocock SJ, Gotzsche PC et al. (2007) The Strengthening the Reporting of Observational Studies in Epidemiology (STROBE) statement: guidelines for reporting observational studies. PLoS Med 4: e296.

26. Altman DG, Schulz KF, Moher D, Egger M, Davidoff F et al. (2001) The revised CONSORT statement for reporting randomized trials: explanation and elaboration. Ann Intern Med 134: 663-694.

27. Bossuyt PM, Reitsma JB, Bruns DE, Gatsonis CA, Glasziou PP et al. (2003) The STARD statement for reporting studies of diagnostic accuracy: explanation and elaboration. Ann Intern Med 138: W1-12.

28. Liberati A, Altman DG, Tetzlaff J, Mulrow C, Gotzsche PC et al. (2009) The PRISMA statement for reporting systematic reviews and meta-analyses of studies that evaluate health care interventions: explanation and elaboration. PLoS Med 6: e1000100.

29. Vandenbroucke JP, von Elm E, Altman DG, Gotzsche PC, Mulrow CD et al. (2007) Strengthening the Reporting of Observational Studies in Epidemiology (STROBE): explanation and elaboration. PLoS Med 4: e297.

30. Sparso T, Grarup N, Andreasen C, Albrechtsen A, Holmkvist J et al. (2009) Combined analysis of 19 common validated type 2 diabetes susceptibility gene variants shows moderate discriminative value and no evidence of gene-gene interaction. Diabetologia 52: 1308-1314.

31. Salinas CA, Koopmeiners JS, Kwon EM, FitzGerald L, Lin DW et al. (2009) Clinical utility of five genetic variants for predicting prostate cancer risk and mortality. Prostate 69: 363-372.

32. Lauenborg J, Grarup N, Damm P, Borch-Johnsen K, Jorgensen T et al. (2009) Common Type 2 Diabetes Risk Gene Variants Associate with Gestational Diabetes. J Clin Endocrinol Metab 94: 145-150.

33. Lyssenko V, Jonsson A, Almgren P, Pulizzi N, Isomaa B et al. (2008) Clinical risk factors, DNA variants, and the development of type 2 diabetes. N Engl J Med 359: 2220-2232.

34. Weedon MN, McCarthy MI, Hitman G, Walker M, Groves CJ et al. (2006) Combining information from common type 2 diabetes risk polymorphisms improves disease prediction. PLOS Med 3: e374.

35. Morrison AC, Bare LA, Chambless LE, Ellis SG, Malloy M et al. (2007) Prediction of coronary heart disease risk using a genetic risk score: the Atherosclerosis Risk in Communities Study. Am J Epidemiol 166: 28-35.

36. van der Net JB, Janssens ACJW, Defesche JC, Kastelein JJ, Sijbrands EJG et al. (2009) Usefulness of genetic polymorphisms and conventional risk factors to predict coronary heart disease in patients with familial hypercholesterolemia. Am J Cardiol 103: 375-380.

37. Wu X, Lin J, Grossman HB, Huang M, Gu J et al. (2007) Projecting individualized probabilities of developing bladder cancer in white individuals. J Clin Oncol 25: 4974-4981.

38. Wong SS, Wilczynski NL, Haynes RB, Ramkissoonsingh R (2003) Developing optimal search strategies for detecting sound clinical prediction studies in MEDLINE. AMIA Annu Symp Proc 728-732.

39. Wilczynski NL, Haynes RB (2004) Developing optimal search strategies for detecting clinically sound prognostic studies in MEDLINE: an analytic survey. BMC Med 2: 23.

40. Aulchenko YS, Ripatti S, Lindqvist I, Boomsma D, Heid IM et al. (2009) Loci influencing lipid levels and coronary heart disease risk in 16 European population cohorts. Nat Genet 41: 47-55.

41. Janssens ACJW, Van Duijn CM (2008) Genome-based prediction of common diseases: advances and prospects. Hum Mol Genet 17: R166-R173.

42. van Hoek M, Dehghan A, Witteman JC, Van Duijn CM, Uitterlinden AG et al. (2008) Predicting type 2 diabetes based on polymorphisms from genome-wide association studies: a population-based study. Diabetes 57: 3122-3128.

43. Vaxillaire M, Veslot J, Dina C, Proenca C, Cauchi S et al. (2008) Impact of common type 2 diabetes risk polymorphisms in the DESIR prospective study. Diabetes 57: 244-254.

44. Von Elm E, Altman DG, Egger M, Pocock SJ, Gotzsche PC et al. (2007) The Strengthening the Reporting of Observational Studies in Epidemiology (STROBE) statement: guidelines for reporting observational studies. PLoS Med 4: e296.

45. Lango H, Palmer CN, Morris AD, Zeggini E, Hattersley AT et al. (2008) Assessing the combined impact of 18 common genetic variants of modest effect sizes on type 2 diabetes risk. Diabetes 57: 3129-3135.

46. Maller J, George S, Purcell S, Fagerness J, Altshuler D et al. (2006) Common variation in three genes, including a noncoding variant in CFH, strongly influences risk of age-related macular degeneration. Nat Genet 38: 1055-1059.

47. Kathiresan S, Melander O, Anevski D, Guiducci C, Burtt NP et al. (2008) Polymorphisms associated with cholesterol and risk of cardiovascular events. N Engl J Med 358: 1240-1249.

48. Meigs JB, Shrader P, Sullivan LM, McAteer JB, Fox CS et al. (2008) Genotype score in addition to common risk factors for prediction of type 2 diabetes. N Engl J Med 359: 2208-2219.

49. Wain HM, Bruford EA, Lovering RC, Lush MJ, Wright MW et al. (2002) Guidelines for human gene nomenclature. Genomics 79: 464-470.

50. Sherry ST, Ward MH, Kholodov M, Baker J, Phan L et al. (2001) dbSNP: the NCBI database of genetic variation. Nucleic Acids Res 29: 308-311.

51. Plat AW, Stoffers HEJH, Klungel OH, van Schayck CP, de Leeuw PW et al. (2009) The contribution of six polymorphisms to cardiovascular risk in a Dutch high-risk primary care population: the HIPPOCRATES project. J Hum Hypertens 23: 659-667.

52. Talmud PJ, Hingorani AD, Cooper JA, Marmot MG, Brunner EJ et al. (2010) Utility of genetic and non-genetic risk factors in prediction of type 2 diabetes: Whitehall II prospective cohort study. BMJ 340: b4838.

53. Pepe MS, Janes HE (2008) Gauging the performance of SNPs, biomarkers, and clinical factors for predicting risk of breast cancer. J Natl Cancer Inst 100: 978-979.

54. Podgoreanu MV, White WD, Morris RW, Mathew JP, Stafford-Smith M et al. (2006) Inflammatory gene polymorphisms and risk of postoperative myocardial infarction after cardiac surgery. Circulation 114: I-275.

55. Humphries SE, Cooper JA, Talmud PJ, Miller GJ (2007) Candidate gene genotypes, along with conventional risk factor assessment, improve estimation of coronary heart disease risk in healthy UK men. Clin Chem 53: 8-16.

56. Paynter NP, Chasman DI, Buring JE, Shiffman D, Cook NR et al. (2009) Cardiovascular disease risk prediction with and without knowledge of genetic variation at chromosome 9p21.3. Ann Intern Med 150: 65-72.

57. Lin X, Song K, Lim N, Yuan X, Johnson T et al. (2009) Risk prediction of prevalent diabetes in a Swiss population using a weighted genetic score--the CoLaus Study. Diabetologia 52: 600-608.

58. Ioannidis JP (2008) Why most discovered true associations are inflated. Epidemiology 19: 640-648.

59. Bleeker SE, Moll HA, Steyerberg EW, Donders AR, Derksen-Lubsen G et al. (2003) External validation is necessary in prediction research: a clinical example. J Clin Epidemiol 56: 826-832.

60. Altman DG, Vergouwe Y, Royston P, Moons KG (2009) Prognosis and prognostic research: validating a prognostic model. BMJ 338: b605.

61. Royston P, Moons KG, Altman DG, Vergouwe Y (2009) Prognosis and prognostic research: Developing a prognostic model. BMJ 338: b604.

62. Janes H, Pepe MS, Gu W (2008) Assessing the value of risk predictions by using risk stratification tables. Ann Intern Med 149: 751-760.

63. Steyerberg EW, Vickers AJ, Cook NR, Gerds T, Gonen M et al. (2010) Assessing the Performance of Prediction Models: A Framework for Traditional and Novel Measures. Epidemiology 21: 128-138.

64. Pepe MS, Feng Z, Huang Y, Longton G, Prentice R et al. (2008) Integrating the predictiveness of a marker with its performance as a classifier. Am J Epidemiol 167: 362-368.

65. Janssens ACJW, Khoury MJ (2010) Assessment of improved prediction beyond traditional risk factors: when does a difference make a difference? Circ: Cardiovasc Genet 3: 3-5.

66. Cook NR, Ridker PM (2009) Advances in measuring the effect of individual predictors of cardiovascular risk: the role of reclassification measures. Ann Intern Med 150: 795-802.

67. Zheng SL, Sun J, Wiklund F, Smith S, Stattin P et al. (2008) Cumulative association of five genetic variants with prostate cancer. N Engl J Med 358: 910-919.

68. Oxman AD, Guyatt GH (1992) A consumer's guide to subgroup analyses. Ann Intern Med 116: 78-84.

69. Little RJA, Rubin DB (1987) Statistical analysis with missing data. New York: John Wiley & Sons.

70. Ioannidis JP, Loy EY, Poulton R, Chia KS (2009) Researching genetic versus nongenetic determinants of disease: a comparison and proposed unification. Sci Transl Med 1: 7ps8.

71. Smith GD, Lawlor DA, Harbord R, Timpson N, Day I et al. (2007) Clustered environments and randomized genes: a fundamental distinction between conventional and genetic epidemiology. PLoS Med 4: e352.

72. Purcell SM, Wray NR, Stone JL, Visscher PM, O'Donovan MC et al. (2009) Common polygenic variation contributes to risk of schizophrenia and bipolar disorder. Nature 460: 748-752.

73. Ioannidis JP (2005) Why most published research findings are false. PLoS Med 2: e124.

74. Ioannidis JP (2007) Limitations are not properly acknowledged in the scientific literature. J Clin Epidemiol 60: 324-329.

75. Horton R (2002) The hidden research paper. JAMA 287: 2775-2778.

76. Docherty M, Smith R (1999) The case for structuring the discussion of scientific papers. BMJ 318: 1224-1225.

77. Clarke M, Chalmers I (1998) Discussion sections in reports of controlled trials published in general medical journals: islands in search of continents? JAMA 280: 280-282.

78. Lumbreras B, Parker LA, Porta M, Pollan M, Ioannidis JP et al. (2009) Overinterpretation of clinical applicability in molecular diagnostic research. Clin Chem 55: 786-794.

79. Gulcher J, Stefansson K (2010) Genetic risk information for common diseases may indeed be already useful for prevention and early detection. Eur J Clin Invest 40: 56-63.

80. Liu F, van Duijn K, Vingerling JR, Hofman A, Uitterlinden AG et al. (2009) Eye color and the prediction of complex phenotypes from genotypes. Curr Biol 19: R192-R193.

81. Yu W, Liu T, Valdez R, Gwinn M, Khoury MJ (2010) Application of support vector machine modeling for prediction of common diseases: the case of diabetes and pre-diabetes. BMC Med Inform Decis Mak 10: 16.

82. Ioannidis JP, Allison DB, Ball CA, Coulibaly I, Cui X et al. (2009) Repeatability of published microarray gene expression analyses. Nat Genet 41: 149-155.

83. Mailman MD, Feolo M, Jin Y, Kimura M, Tryka K et al. (2007) The NCBI dbGaP database of genotypes and phenotypes. Nat Genet 39: 1181-1186.

84. Manolio TA, Rodriguez LL, Brooks L, Abecasis G, Ballinger D et al. (2007) New models of collaboration in genome-wide association studies: the Genetic Association Information Network. Nat Genet 39: 1045-1051.

85. Rochon PA, Hoey J, Chan AW, Ferris LE, Lexchin J et al. (2010) Financial conflicts of interest checklist 2010 for clinical research studies. Open Medicine 4: e70.

86. Vandenbroucke JP (2009) STREGA, STROBE, STARD, SQUIRE, MOOSE, PRISMA, GNOSIS, TREND, ORION, COREQ, QUOROM, REMARK... and CONSORT: for whom does the guideline toll? J Clin Epidemiol 62: 594-596.

87. Moher D, Schulz KF, Simera I, Altman DG (2010) Guidance for developers of health research reporting guidelines. PLoS Med 7: e1000217.

88. Smidt N, Rutjes AW, van der Windt DA, Ostelo RW, Bossuyt PM et al. (2006) The quality of diagnostic accuracy studies since the STARD statement: has it improved? Neurology 67: 792-797.

89. Hopewell S, Dutton S, Yu L, Chan A, Altman DG (2010) The quality of reports of randomised trials in 2000 and 2006: comparative study of articles indexed in PubMed. BMJ 340: c723.

90. Altman DG, Simera I, Hoey J, Moher D, Schulz K (2008) EQUATOR: reporting guidelines for health research. Lancet 371: 1149-1150.

91. Cook NR (2007) Use and misuse of the receiver operating characteristic curve in risk prediction. Circulation 115: 928-935.

92. Balkau B, Lange C, Fezeu L, Tichet J, Lauzon-Guillain B et al. (2008) Predicting diabetes: clinical, biological, and genetic approaches: data from the Epidemiological Study on the Insulin Resistance Syndrome (DESIR). Diabetes Care 31: 2056-2061.

93. Lyssenko V, Almgren P, Anevski D, Orho-Melander M, Sjogren M et al. (2005) Genetic prediction of future type 2 Diabetes. PLOS Med 2: e345.

94. Beekman M, Nederstigt C, Suchiman HE, Kremer D, van der BR et al. (2010) Genome-wide association study (GWAS)-identified disease risk alleles do not compromise human longevity. Proc Natl Acad Sci U S A 107: 18046-18049.

95. Janssens ACJW, Aulchenko YS, Elefante S, Borsboom GJJM, Steyerberg EW et al. (2006) Predictive testing for complex diseases using multiple genes: fact or fiction? Genet Med 8: 395-400.

**Acknowledgments**

The findings and conclusions in this report are those of the authors and do not necessarily reflect the views of the Department of Health and Human Services.

**Author contributions**

All authors were participants at the workshop held on 16-17 December 2009 in Atlanta GA, USA. ACJWJ and MJK initiated the GRIPS Initiative. ACJWJ wrote the first draft of the checklist and the paper, which was reviewed by JPAI, CMvD, JL and MJK and then by all workgroup participants. MJK was responsible for the coordination of the meeting.

**Abbreviations**

Abbreviations used in the examples are not listed here.

AUC: Area Under the receiver operating characteristic Curve

CONSORT: Consolidated Standard of Reporting Trials

dbGaP: Database of Genotype and Phenotype

EQUATOR: Enhancing the QUAlity and Transparency Of health Research

GAIN: Genetic Association Information Network

GRIPS: Genetic RIsk Prediction Studies

HuGENet: Human Genome Epidemiology Network

IDI: Integrated Discrimination Improvement

MeSH: Medical Subject Headings

REMARK: Guidelines for Reporting of tumor MARKer studies

SNP: single nucleotide polymorphism

STARD: STAndards for Reporting Diagnostic accuracy

STREGA: STrenghtening the REporting of Genetic Association studies

STROBE: Strengthening the Reporting of OBservational studies in Epidemiology

**Table 1.** Reporting recommendations for evaluations of risk prediction models that include genetic variants.

| **TITLE & ABSTRACT** | | |
| --- | --- | --- |
|  | 1 | (a) Identify the article as a study of risk prediction using genetic factors. (b) Use recommended keywords in the abstract: genetic or genomic, risk, prediction. |
| **INTRODUCTION** | | |
| Background and Rationale | 2 | Explain the scientific background and rationale for the prediction study. |
| Objectives | 3 | Specify the study objectives and state the specific model(s) that is/are investigated. State if the study concerns the development of the model(s), a validation effort, or both. |
| **METHODS** | | |
| Study design and setting | 4* | Specify the key elements of the study design and describe the setting, locations and relevant dates, including periods of recruitment, follow-up and data collection. |
| Participants | 5* | Describe eligibility criteria for participants, and sources and methods of selection of participants. |
| Variables: definition | 6* | Clearly define all participant characteristics, risk factors and outcomes. Clearly define genetic variants using a widely-used nomenclature system. |
| Variables: assessment | 7* | (a) Describe sources of data and details of methods of assessment (measurement) for each variable. (b) Give a detailed description of genotyping and other laboratory methods. |
| Variables: coding | 8 | (a) Describe how genetic variants were handled in the analyses. (b) Explain how other quantitative variables were handled in the analyses. If applicable, describe which groupings were chosen, and why. |
| Analysis: risk model construction | 9 | Specify the procedure and data used for the derivation of the risk model. Specify which candidate variables were initially examined or considered for inclusion in models. Include details of any variable selection procedures and other model-building issues. Specify the horizon of risk prediction (e.g., 5-year risk). |
| Analysis: validation | 10 | Specify the procedure and data used for the validation of the risk model. |
| Analysis: missing data | 11 | Specify how missing data were handled. |
| Analysis: statistical methods | 12 | Specify all measures used for the evaluation of the risk model including, but not limited to, measures of model fit and predictive ability. |
| Analysis: other | 13 | Describe all subgroups, interactions and exploratory analyses that were examined. |
| **RESULTS** | | |
| Participants | 14* | Report the numbers of individuals at each stage of the study. Give reasons for non-participation at each stage. Report the number of participants not genotyped, and reasons why they were not genotyped. |
| Descriptives: population | 15* | Report demographic and clinical characteristics of the study population, including risk factors used in the risk modeling. |
| Descriptives: model estimates | 16 | Report unadjusted associations between the variables in the risk model(s) and the outcome. Report adjusted estimates and their precision from the full risk model(s) for each variable. |
| Risk distributions | 17* | Report distributions of predicted risks and/or risk scores. |
| Assessment | 18 | Report measures of model fit and predictive ability, and any other performance measures, if pertinent. |
| Validation | 19 | Report any validation of the risk model(s). |
| Other analyses | 20 | Present results of any subgroup, interaction or exploratory analyses, whenever pertinent. |
| **DISCUSSION** | | |
| Limitations | 21 | Discuss limitations and assumptions of the study, particularly those concerning study design, selection of participants, measurements and analyses, and discuss their impact on the results of the study. |
| Interpretation | 22 | Give an overall interpretation of results considering objectives, limitations, multiplicity of analyses, results from similar studies, and other relevant evidence. |
| Generalizability | 23 | Discuss the generalizability and, if pertinent, the health care relevance of the study results. |
| **OTHER** | | |
| Supplementary information | 24 | State whether databases for the analyzed data, risk models and/or protocols are or will become publicly available and if so, how they can be accessed. |
| Funding | 25 | Give the source of funding and the role of the funders for the present study. State whether there are any conflicts of interest. |

* Marked items should be reported for every population in the study.

**Table 2.** Genetic terminology used in titles of genetic risk prediction studies and retrieval of the studies in PubMed

| Terminology | Reference | PubMed Clinical Query | | | | Genetic risk prediction^2^ |
| --- | --- | --- | --- | --- | --- | --- |
|  |  | Clinical Prediction Guides | | Prognosis | |  |
|  |  | Narrow | Broad | Narrow | Broad |  |
| Candidate gene genotypes | [55] | No | Yes | No | Yes | No |
| DNA variants | [33] | No | Yes | No | Yes | Yes |
| Gene polymorphisms | [54] | No | Yes | Yes | Yes | Yes |
| Gene variants | [32] | No | No | Yes | Yes | No |
| Genetic approaches | [92] | No | Yes | No | Yes | Yes |
| Genetic prediction | [93] | No | Yes | No | Yes | Yes |
| Genetic risk factors | [52] | No | Yes | Yes | Yes | Yes |
| Genetic risk score | [35] | No | Yes | Yes | Yes | Yes |
| Genetic variables | [6] | No | Yes | No | Yes | Yes |
| Genetic variants | [67]  [31] | No  No | No  Yes | No  Yes | No  Yes | No  Yes |
| Genetic variation | [56] | No | Yes | Yes | Yes | Yes |
| Genotype score | [48] | No | Yes | No | Yes | Yes |
| Molecular prediction | [7] | No | Yes | Yes | Yes | Yes |
| Polygenic determinants | [8] | No | No | No | No | No |
| Polymorphisms | [51]  [42]  [47]  [34]  [43]  [36] | No  No  No  No  No  No | Yes  Yes  Yes  Yes  Yes  Yes | No  No  Yes  No  Yes  No | Yes  Yes  Yes  Yes  Yes  Yes | Yes  Yes  No  Yes  Yes  Yes |
| Susceptibility gene variants | [30] | No | No | No | No | No |
| Weighted genetic score | [57] | No | No | No | Yes | Yes |
| No mention of genetics in title | [37] | Yes | Yes | No | Yes | Yes |
|  |  |  |  |  |  |  |
| Retrieved (out of 24) | | 1 | 19 | 9 | 21 | 18 |
| (genetic[ti] or gene[ti] or DNA[ti] or polymorphism*[ti] or molecular[ti] or polygenic[ti]) AND <query>^1^ | | 4,772 | 156,641 | 18,090 | 67,931 |  |
| (Genetic or genomic) risk prediction^2^ | |  |  |  |  | 1,597 |

Retrieval data were obtained from PubMed queries conducted in February 2010. ^1^ The first part of this strategy captures the genetic descriptions from the titles of all papers listed in the table, except the one that had no mention of genetics in the title. The second part refers to the query listed in the column heading. ^2^ The search strategy used in the last column is described in the last row.

**Table 3.** Example Table: Description of genetic variants used in the analyses. Adapted from [48].

| **SNP** | **Locus** | **Chromosome** | **Locus relative to gene** | **Risk allele** | **Source** |
| --- | --- | --- | --- | --- | --- |
| rs10923931 | *NOTCH2* | 1 | Intron 5 | T | Zeggini et al. |
| rs10490072 | *BCL11A* | 2 | 3' of gene | T | Zeggini et al. |
| rs7578597 | *THADA* | 2 | Missense,exon 24 | T | Zeggini et al. |
| rs1470579 | *IGF2BP2* | 3 | Intron 2 | C | Saxena et al. |
| rs1801282 | *PPARg* | 3 | Intron 1 | C | Saxena et al. |
| rs4607103 | *ADAMTS9* | 3 | Intron 2 | C | Zeggini et al. |
| rs7754840 | *CDKAL1* | 6 | Intron 5 | C | Saxena et al. |
| rs9472138 | *VEGFA* | 6 | 3' of gene | T | Zeggini et al. |
| rs864745 | *JAZF1* | 7 | Intron 1 | T | Zeggini et al. |
| rs13266634 | *SLC30A8* | 8 | Missense, exon 8 | C | Saxena et al. |
| rs10811661 | *CDKNA/2B* | 9 | 5' of gene | T | Saxena et al. |
| rs1111875 | *HHEX* | 10 | 3' of gene | C | Saxena et al. |
| rs12779790 | *CDC123,CAMK1D* | 10 | 3' of gene | G | Zeggini et al. |
| rs7903146 | *TCF7L2* | 10 | Intron 6 | T | Saxena et al. |
| rs5219 | *KCNJ11* | 11 | Missense, exon 1 | T | Saxena et al. |
| rs689 | *INS* | 11 | Intron 1 | T | Meigs et al. |
| rs1153188 | *DCD* | 12 | 5' of gene | A | Zeggini et al. |
| rs7961581 | *TSPAN8, LGR5* | 12 | 5' of gene | C | Zeggini et al. |

**Table 4.** Example Table: Demographic and clinical characteristics of study participants with and without prostate cancer. Adapted from [31].

| Characteristic | Cases |  | Controls |  |
| --- | --- | --- | --- | --- |
|  | N=1,308 | % | N=1,266 | % |
| Age (years) |  |  |  |  |
| 35–49 | 102 | 7.8 | 107 | 8.5 |
| 50–54 | 188 | 14.4 | 178 | 14.1 |
| 55–59 | 325 | 24.9 | 343 | 27.1 |
| 60–64 | 395 | 30.2 | 334 | 26.4 |
| 65–69 | 153 | 11.7 | 160 | 12.6 |
| 70–74 | 145 | 11.1 | 144 | 11.4 |
| 1^st^ degree family history of prostate cancer |  |  |  |  |
| No | 1,025 | 78.4 | 1,125 | 88.9 |
| Yes | 283 | 21.6 | 141 | 11.1 |
| PSA at diagnosis or interview (ng/ml) |  |  |  |  |
| 0–3.9 | 178 | 13.6 | 351 | 27.7 |
| 4.0–9.9 | 721 | 55.1 | 33 | 2.6 |
| 10.0–19.9 | 190 | 14.5 | 6 | 0.5 |
| ≥20.0 | 118 | 9.0 | 0 | — |
| Gleason score |  |  |  |  |
| 2–4 | 66 | 5.1 |  |  |
| 5–6 | 681 | 52.2 |  |  |
| 7=3+4 | 355 | 27.2 |  |  |
| 7=4+3 | 76 | 5.8 |  |  |
| 8–10 | 126 | 9.7 |  |  |
| Primary treatment |  |  |  |  |
| Radical prostatectomy | 770 | 58.9 |  |  |
| Radiation | 352 | 26.9 |  |  |
| Androgen deprivation therapy | 60 | 4.6 |  |  |
| Other treatment | 11 | 0.8 |  |  |
| Active surveillance | 115 | 8.8 |  |  |

**Table 5.** Example Table: Descriptive associations between demographic, environmental and genetic variables and progression to advanced age-related macular degeneration. Adapted from [6].

|  | **Progressors,** | **Nonprogressors,** |  |  |
| --- | --- | --- | --- | --- |
|  | ***n (*%)** | ***n (*%)** | **OR (95%CI)** | ***P*** |
| Total patients | 279 | 1167 |  |  |
| Age (y) |  |  |  |  |
| <70 | 137 (49) | 743 (64) | 1.0 |  |
| 70+ | 142 (51) | 424 (36) | 1.8 (1.4–2.4) | <0.001 |
| Sex |  |  |  |  |
| Female | 163 (58) | 694 (59) | 1.0 | 0.74 |
| Male | 116 (42) | 473 (41) | 1.0 (0.8–1.4) |  |
| Education |  |  |  |  |
| ≤High school | 119 (43) | 383 (33) | 1.0 | 0.002 |
| >High school | 160 (57) | 784 (67) | 0.7 (0.5–0.9) |  |
| Baseline AMD Grades |  |  |  |  |
| 2 | 8 (3) | 446 (38) | 1.0 |  |
| 3 | 161 (58) | 566 (48) | 15.9 (7.7–32.6) | <0.001 |
| 4 | 110 (39) | 155 (13) | 39.6 (18.9–83.0) |  |
| Smoking |  |  |  |  |
| Never | 110 (39) | 557 (48) | 1.0 |  |
| Past | 137 (49) | 564 (48) | 1.2 (0.9–1.6) | 0.14 |
| Current | 32 (11) | 46 (4) | 3.5 (2.1–5.8) | <0.001 |
| BMI |  |  |  |  |
| <25 | 69 (25) | 416 (36) | 1.0 |  |
| 25–29 | 130 (47) | 484 (41) | 1.6 (1.2–2.2) | 0.003 |
| 30+ | 80 (29) | 267 (23) | 1.8 (1.3–2.6) | 0.001 |
| Treatment Group |  |  |  |  |
| Placebo | 74 (27) | 264 (23) | 1.0 |  |
| Antioxidants | 77 (28) | 295 (25) | 0.9 (0.7–1.3) | 0.70 |
| Zinc | 67 (24) | 294 (25) | 0.8 (0.6–1.2) | 0.27 |
| Antioxidants and zinc | 61 (22) | 314 (27) | 0.7 (0.5–1.0) | 0.056 |
| rs1061170 |  |  |  |  |
| TT | 39 (14) | 366 (31) | 1.0 |  |
| CT | 116 (42) | 521 (45) | 2.1 (1.4–3.1) |  |
| CC | 124 (44) | 280 (24) | 4.1 (2.8–6.1) | <0.001 |
| rs10490924 |  |  |  |  |
| GG | 67 (24) | 612 (52) | 1.0 |  |
| GT | 138 (49) | 446 (38) | 2.8 (2.1–3.9) | <0.001 |
| TT | 74 (27) | 109 (9) | 6.2 (4.2–9.1) |  |
| rs1410996 |  |  |  |  |
| TT | 8 (3) | 158 (14) | 1.0 |  |
| CT | 74 (27) | 472 (40) | 3.1 (1.5–6.6) | <0.001 |
| CC | 197 (71) | 537 (46) | 7.2 (3.5–15.0) |  |
| rs9332739 |  |  |  |  |
| GG | 271 (97) | 1075 (92) | 1.0 |  |
| CG/CC | 8 (3) | 92 (8) | 0.3 (0.2–0.7) | 0.005 |
| rs641153 |  |  |  |  |
| CC | 256 (92) | 1023 (88) | 1.0 |  |
| CT/TT | 23 (8) | 143 (12) | 0.6 (0.4–1.0) | 0.06 |
| rs2230199 |  |  |  |  |
| CC | 124 (44) | 652 (56) | 1.0 |  |
| CG | 130 (47) | 456 (39) | 1.5 (1.1–2.0) |  |
| GG | 25 (9) | 59 (5) | 2.2 (1.3–3.7) | <0.001 |

**Table 6.** Example Table: Multivariate association between demographic, environmental, and genetic risk factors and progression to advanced age-related macular degeneration (AMD). Adapted from [6].

|  | Regression Coefficient (β_i_) | OR (95% CI)* | ***P*** |
| --- | --- | --- | --- |
| Intercept (α) | -5.780 |  |  |
| Age (y) |  |  |  |
| ≤70 | 0 | 1.0 |  |
| >70 | 0.4116 | 1.5 (1.1–2.0) | 0.008 |
| Sex |  |  |  |
| Female | 0 | 1.0 |  |
| Male | 0.0688 | 1.1 (0.8–1.5) | 0.68 |
| Education |  |  |  |
| ≤High school | 0 | 1.0 |  |
| >High school | -0.1280 | 0.9 (0.6–1.2) | 0.42 |
| Baseline Grade |  |  |  |
| 2 | 0 | 1.0 |  |
| 3 | 2.3944 | 11.0 (5.3–22.8) | <0.001 |
| 4 | 2.9521 | 19.1 (8.9–41.2) | <0.001 |
| Smoking |  |  |  |
| Never | 0 | 1.0 |  |
| Past | 0.1211 | 1.1 (0.8–1.6) | 0.47 |
| Current | 1.1261 | 3.1 (1.7–5.6) | <0.001 |
| BMI |  |  |  |
| <25 | 0 | 1.0 |  |
| 25–29 | 0.5170 | 1.7 (1.2–2.4) | 0.006 |
| 30+ | 0.4754 | 1.6 (1.1–2.4) | 0.024 |
| Treatment Group |  |  |  |
| Placebo | 0 | 1.0 |  |
| Antioxidants | -0.1299 | 0.9 (0.6–1.3) | 0.54 |
| Zinc | -0.3897 | 0.7 (0.4–1.0) | 0.075 |
| Antioxidants and zinc | -0.4973 | 0.6 (0.4–0.9) | 0.023 |
| rs1061170 |  |  |  |
| TT | 0 | 1.0 |  |
| CT | 0.2644 | 1.3 (0.8–2.1) | 0.29 |
| CC | 0.6778 | 2.0 (1.1–3.5) | 0.019 |
| *P* trend |  |  | 0.014 |
| rs10490924 |  |  |  |
| GG | 0 | 1.00 |  |
| GT | 0.8396 | 2.3 (1.6–3.3) | <0.001 |
| TT | 1.3837 | 4.0 (2.6–6.1) | <0.001 |
| *P* trend |  |  | <0.001 |
| rs1410996 |  |  |  |
| TT | 0 | 1.0 |  |
| CT | 0.5251 | 1.7 (0.7–4.0) | 0.23 |
| CC | 0.8606 | 2.4 (1.0–5.8) | 0.061 |
| *P* trend |  |  | 0.029 |
| rs9332739 |  |  |  |
| GG | 0 | 1.0 |  |
| CG/CC | -1.0510 | 0.4 (0.2–0.8) | 0.010 |
| rs641153 |  |  |  |
| CC | 0 | 1.0 |  |
| CT or TT | -0.2147 | 0.8 (0.5–1.4) | 0.42 |
| rs2230199 |  |  |  |
| CC | 0 | 1.0 |  |
| CG | 0.3679 | 1.4 (1.1–2.0) | 0.022 |
| GG | 0.5970 | 1.8 (1.0–3.2) | 0.044 |
| *P* trend |  |  | 0.006 |

* ORs adjusted for age (<70, ≥70), sex, education (≤high school, >high school), smoking (never, past, current), baseline AMD grade, BMI (<25, 25–29, 30+), and treatment groups (placebo, antioxidants, zinc, and antioxidants plus zinc), and all six genetic variants and associated genotypes as listed in the table. Calculation of the AMD Progression Risk Score = α + β_i_X_i_, where i refers to each of the variables listed.

**Table 7.** Example Table: Net reclassification improvement based on addition of gene count score to Framingham offspring risk score. Adapted from [52].

| Framingham offspring risk score | Framingham offspring risk score  plus gene count score | | | | Reclassified | | Net correctly reclassified |
| --- | --- | --- | --- | --- | --- | --- | --- |
|  | <5% | 5-10% | 10-15% | >15% | Increased risk | Decreased risk |  |
| People without diabetes during follow-up | | | | | | | |
| <5% | 2295 | 48 | 0 | 0 |  |  |  |
| 5-10% | 36 | 482 | 43 | 0 | 121 | 64 | −1.7% |
| 10-15% | 0 | 19 | 181 | 30 |  |  |  |
| >15% | 0 | 0 | 9 | 181 |  |  |  |
| People with diabetes during follow-up | | | | | | | |
| <5% | 52 | 8 | 0 | 0 |  |  |  |
| 5-10% | 2 | 37 | 3 | 0 | 14 | 11 | 1.5% |
| 10-15% | 0 | 4 | 24 | 3 |  |  |  |
| >15% | 0 | 0 | 5 | 64 |  |  |  |
| Net reclassification improvement -0.2% (95% CI −5.1 to 4.7); P=0.94. | | | | | | | |

**Figure 1.** Example: Distribution of the number of disease risk alleles among sporadic long-lived participants of the Leiden 85 Plus Study and Netherlands Twin Register controls [94].


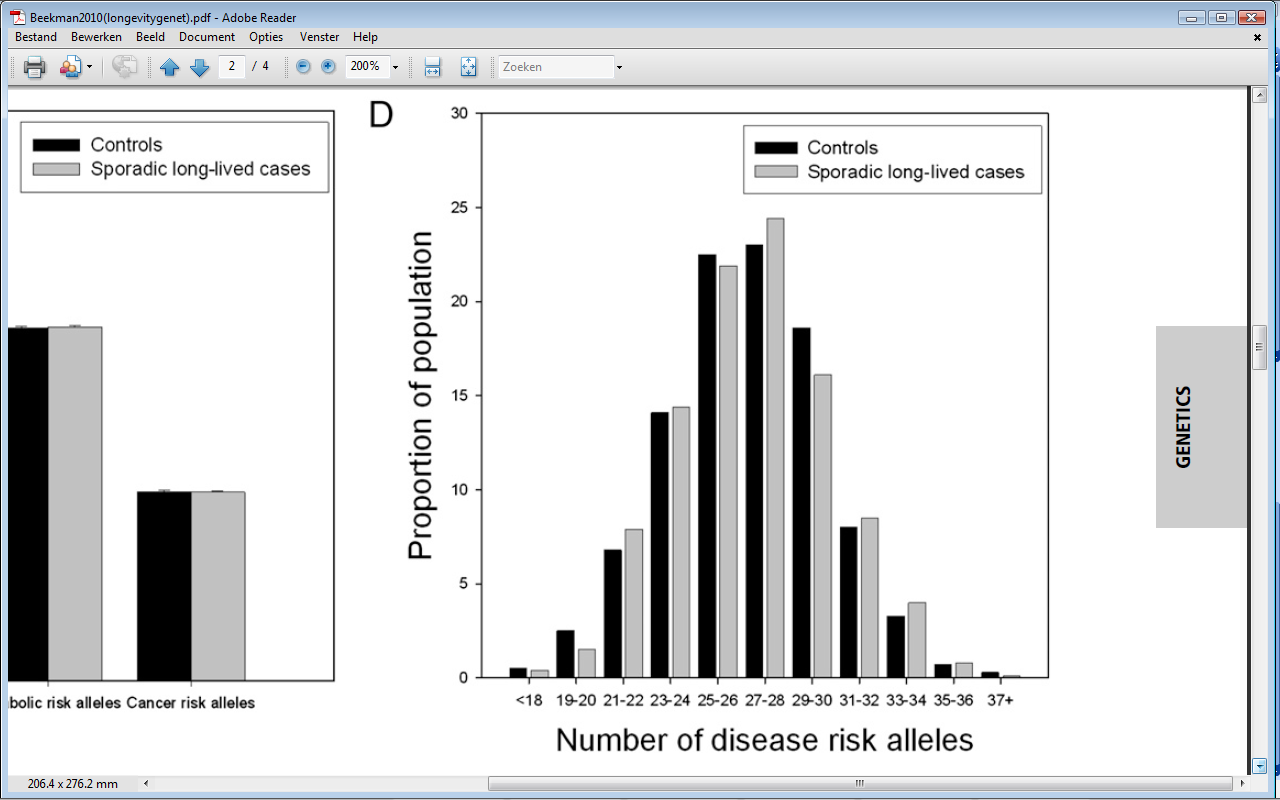


**Figure 2.** Example: ROC curve analysis of adding genetic variables to clinical risk factors for the prediction of age-related macular degeneration.


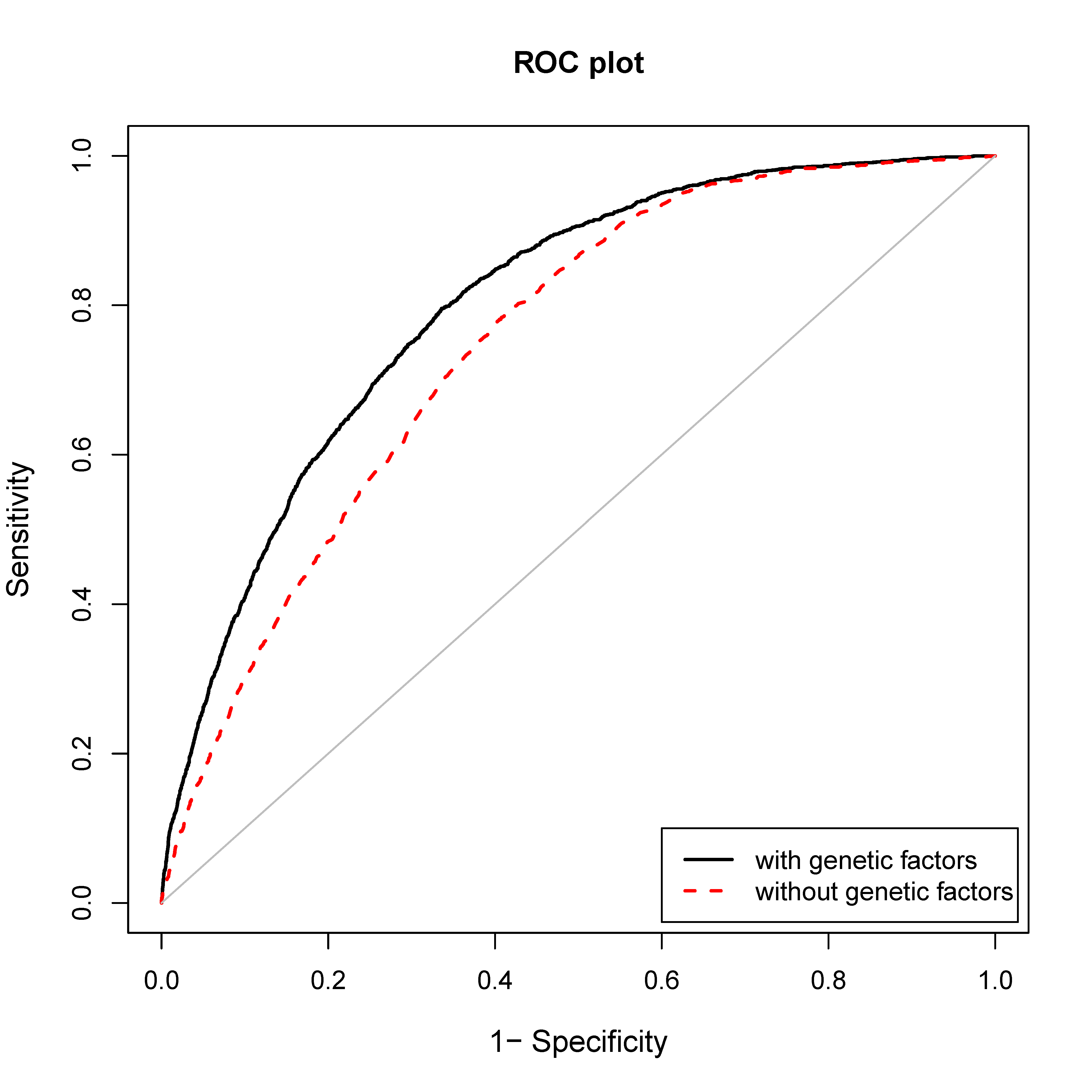


Area under the receiver operating characteristic curve for the age-related macular degeneration (AMD). The risk models were constructed from published genotype/exposure frequencies and odds ratios [6], using a simulation method that has been described previously [95]. The clinical prediction model was based on age, sex, education, baseline AMD grade, smoking, body mass index and treatment. The added genetic factors were six single nucleotide polymorphisms. The curves indicate the sensitivity and 1-specificity for every possible cut-off value of predicted risks. The diagonal line indicates a hypothetical random predictor, which AUC equals 0.50.
